# RAB7A Regulates Vimentin Phosphorylation through AKT and PAK

**DOI:** 10.3390/cancers13092220

**Published:** 2021-05-06

**Authors:** Roberta Romano, Matteo Calcagnile, Azzurra Margiotta, Lorenzo Franci, Mario Chiariello, Pietro Alifano, Cecilia Bucci

**Affiliations:** 1 Department of Biological and Environmental Sciences and Technologies (DiSTeBA), University of Salento, 73100 Lecce, Italy; roberta.romano@unisalento.it (R.R.); matteo.calcagnile@unisalento.it (M.C.); azzurra.marg@libero.it (A.M.); pietro.alifano@unisalento.it (P.A.); 2 Istituto di Fisiologia Clinica (IFC), Consiglio Nazionale delle Ricerche (CNR), 53100 Siena, Italy; lorenzofranci4@gmail.com (L.F.); mario.chiariel@gmail.com (M.C.); 3 Core Research Laboratory (CRL), Istituto per lo Studio, La Prevenzione e la Rete Oncologica (ISPRO), 53100 Siena, Italy; 4 Department of Medical Biotechnologies, University of Siena, 53100 Siena, Italy

**Keywords:** cell migration, intermediate filaments, RAC1, beta-catenin, NF-kB, cofilin

## Abstract

**Simple Summary:**

RAB7A (RAs-related in Brain 7A) is a master regulator of intracellular traffic controlling transport to late endosomes and lysosomes, two organelles of the endocytic pathway important for degradation. Thanks to this function, RAB7A is also involved in cellular processes linked to cancer, such as apoptosis, cytoskeletal reorganization, and cell migration. Therefore, the interest in the role of RAB7A in cancer progression is increasing. Previously, we demonstrated that RAB7A regulates phosphorylation and assembly of vimentin, a cytoskeletal intermediate filament protein, which is also an important mesenchymal marker of cancer cells. The aim of the present study is the identification of the kinases responsible for vimentin phosphorylation whose activity is affected by the modulation of RAB7A expression. We found that RAB7A is able to regulate AKT (also called protein kinase B or PKB) and PAK1 (P21-Activated Kinase 1) and several of their downstream effectors, which control proliferation, apoptosis, survival, migration, and invasion. These data suggest that RAB7A could have a key role in cancer development.

**Abstract:**

RAB7A is a small GTPase that controls the late endocytic pathway but also cell migration through RAC1 (Ras-related C3 botulinum toxin substrate 1) and vimentin. In fact, RAB7A regulates vimentin phosphorylation at different sites and vimentin assembly, and, in this study, we identified vimentin domains interacting with RAB7A. As several kinases could be responsible for vimentin phosphorylation, we investigated whether modulation of RAB7A expression affects the activity of these kinases. We discovered that RAB7A regulates AKT and PAK1, and we demonstrated that increased vimentin phosphorylation at Ser38 (Serine 38), observed upon RAB7A overexpression, is due to AKT activity. As AKT and PAK1 are key regulators of several cellular events, we investigated if RAB7A could have a role in these processes by modulating AKT and PAK1 activity. We found that RAB7A protein levels affected beta-catenin and caspase 9 expression. We also observed the downregulation of cofilin-1 and decreased matrix metalloproteinase 2 (MMP2) activity upon RAB7A silencing. Altogether these results demonstrate that RAB7A regulates AKT and PAK1 kinases, affecting their downstream effectors and the processes they regulate, suggesting that RAB7A could have a role in a number of cancer hallmarks.

## 1. Introduction

RAB (Ras-related in Brain) proteins are small GTPases that act as molecular switches to regulate many steps of membrane traffic, from the formation of vesicles at donor compartments to their transport, tethering, and fusion to the correct target compartments [1]. RAB proteins cycle between an inactive GDP-bound and an active GTP-bound form interacting with several effector proteins that confer to the RAB proteins the ability to regulate different steps of intracellular trafficking [1]. In particular, RAB7A is a small GTPase located on late endosomes and lysosomes that controls endocytic traffic from early to late endosomes and lysosomes [2,3,4]. In addition, RAB7A is responsible for the biogenesis of lysosomes, phagolysosomes, and autolysosomes [2,3,4], and it is important for endoplasmic reticulum morphology and homeostasis [5], for ER (Endoplasmic Reticulum)-endosomes and mitochondria-lysosomes contact sites [6,7] and for lipid droplet breakdown [8].

Two intermediate filament proteins, peripherin and vimentin interact with RAB7A, which regulates their assembly and phosphorylation state [9,10]. In particular, we have demonstrated that silencing of RAB7A in HeLa cells is able to reduce vimentin phosphorylation at different sites, altering vimentin assembly [9]. In fact, depletion of RAB7A causes a decrease of Ser38 and Ser55 phosphorylated vimentin [9]. According to the fact that phosphorylation of vimentin is responsible for vimentin depolymerization and, thus, increases soluble vimentin, lack of RAB7A caused the reduction of vimentin amount in the soluble pool, while insoluble filamentous vimentin was increased [9]. However, how RAB7A regulates this post-translational modification of vimentin is still unknown.

Phosphorylation of vimentin residues is mediated by several different kinases [11,12,13,14,15,16,17,18]. In particular, it has been shown that AKT1, PKA (Protein Kinase A), PAK1 and ROCK2 (Rho-associated protein kinase 2) phosphorylate vimentin Ser38 residue [11,12,14,15,17]. For instance, the tail region of AKT1 binds the head domain of vimentin determining Ser38 phosphorylation and promoting cell motility and invasion [11]. PAK1 is able to phosphorylate also the Ser55 site of vimentin [13]. Specific phosphorylated sites of these kinases have been associated with their activated forms, which are able to phosphorylate their substrates. For instance, phosphorylation of Ser473 of AKT is necessary for its activation [19] while phosphorylation of Ser1366 reveals the activation status of ROCK2 [20]. A connection between PAK1 and AKT was demonstrated, as PAK kinase acts as a scaffold facilitating AKT activation and its recruitment to the membrane [21]. Vimentin phosphorylation state mediated by a specific kinase has been associated recently with cell adhesion on fibronectin and it has been demonstrated that RAB7A is able to regulate vimentin reorganization and cell adhesion during migration [22,23].

In this study, we defined vimentin domains responsible for the interaction with RAB7A and we investigated the role of RAB7A on the activation state of several kinases that could, in turn, regulate vimentin phosphorylation. We established that RAB7A is able to regulate PAK1 abundance and AKT activation and that AKT could be the key element through which RAB7A modulates vimentin phosphorylation state and possibly cell adhesion.

## 2. Materials and Methods

### 2.1. Cell Lines and Chemicals

HeLa (ATCC CCL-2; human cervix adenocarcinoma) and NCI H1299 (ATCC CRL-5803; human lung carcinoma) cells were grown in Dulbecco’s modified Eagle medium (DMEM) containing 10% FBS (Fetal Bovine Serum), 2 mM L-glutamine, 100 U/mL penicillin, and 10 mg/mL streptomycin in an incubator at 37 °C, under 5% CO_2_. Cells were confirmed to be contamination-free, and their identity was confirmed by short-tandem repeat profiling.

AKT inhibitor GDC-0068 was supplied by Cayman Chemical (Ann Arbor, MI, USA). Other chemicals were from Sigma-Aldrich (St Louis, MO, USA). Tissue culture reagents were from Sigma-Aldrich or Gibco (Waltham, MA, USA).

### 2.2. Mutagenesis and Plasmids Construction

Plasmids encoding pcDNA3_2XHA and pcDNA3_2XHA-RAB7A wild type have been described previously [24]. For the expression of hemagglutinin (HA)-tagged vimentin, a pCMV6-AN-HA-vimentin was used [9]. The plasmid encoding myc-vimentin was obtained from OriGene (RC201546). Plasmids encoding deleted forms of vimentin were created by amplifying portions of the wild type vimentin ORF (Open Reading Frame) with specific primers and by using AsiSI and MluI restriction enzymes to replace the wild type form in the pCMV6-myc plasmid. For vimentin deletion constructs the following oligonucleotides were used: vimentin 1-256, 5′-TCTGCCGCCGCGATCGCCATGTCCACCAGG-3′ and 5′-CGTACGCGTGATTTGGACATGCTGTTC-3′; vimentin 92-256, 5′-ATAGCGATCGCCATGATCAACACCGAG-3′ and 5′-CGTACGCGTGATTTGGACATGCTGTTC-3′; vimentin 256-411, 5′-ATAGCGATCGCCATGATCGATGTG-3′ and 5′-CGTACGCGTAATCCTGCTCTCCTC-3′; vimentin 256-466, 5′-ATAGCGATCGCCATGATCGATGTG-3′ and 5′-CCGCGTACGCGTTTCAAGGTCATC-3′; vimentin 1-141, 5′-TCTGCCGCCGCGATCGCCATGTCCACCAGG-3′ and 5′-CTGATTACGCGTTTGGCCCTTGAGCTGCTC-3′; vimentin 142-256, 5′-TAAATAGCGATCGCCATGGGCAAGTCGCGCCCTGGG-3′ and 5′-CGTACGCGTGATTTGGACATGCTGTTC-3′. RAB7A^Y183F^ and RAB7A^Y183H^ plasmids were obtained by PCR-mediated mutagenesis using the QuickChange XL Site-Directed Mutagenesis Kit (Stratagene, San Diego, CA, USA). The mutagenesis was performed on RAB7A wildtype cDNA previously cloned in pcDNA3_2XHA, in frame with DNA coding for HA tag. Oligonucleotides used were 5′-GAAACAGAGGTGGAGCTGTTCAATGAATTCCCTGAACC-3′ and 5′-GGTTCAGGGAATTCATTGAACAGCTCCACCTCTGTTTC-3′ for RAB7A^Y183F^ and 5′-ACAGAGGTGGAGCTGCACAATGAATTCCCTG-3′ and 5′-CAGGGAATTCATTGTGCAGCTCCACCTCTGT-3′ for RAB7A^Y183H^. For the luciferase assays, we used the vector pCEFL-AU5 RasV12 and a reporter plasmid containing five consensus NF-kB (Nuclear Factor Kappa B) responsive sites followed by the luciferase gene (Stratagene), as previously described [25]. All the newly made constructs were sequence verified.

### 2.3. Antibodies

Primary antibodies used in this work were rabbit polyclonal anti-HA (ab9110) from Abcam (Cambridge, UK); mouse monoclonal anti-RAB7A (sc376362), anti-vimentin (sc6260), anti-p-αPAK (sc-135755), anti-αPAK (sc-166887), anti-cofilin-1 (sc-53934), anti-caspase 9 (sc-56073), rabbit polyclonal anti-myc (sc-789), anti-p-AKT 1/2/3 (sc7985-R), anti-AKT 1/2/3 (sc8312), anti-p-PKA α/β/γ cat (sc32968), anti-PKA (sc903), and ROCK-2 (sc5561), from Santa Cruz Biotechnology (Santa Cruz, CA, USA); mouse monoclonal anti-β-catenin (610154) from BD Transduction Laboratories (San Jose, CA, USA); rabbit polyclonal anti-cleaved caspase 9 (#7237), anti-NF-kB p65 (#8242), anti-matrix metalloproteinase 2 (MMP2) (#4022), anti-p-vimentin S38 (#13614), and anti-p-p65/RelA (Ser536) (#3033) from Cell Signaling Technology (Leiden, The Netherlands); mouse monoclonal anti-tubulin (T5168) from Sigma-Aldrich and rabbit polyclonal p-ROCK2 (GTX122651) from GeneTex (Irvine, CA, USA). Secondary antibodies conjugated to fluorochromes (for immunofluorescence analyses) or horseradish peroxidase (HRP, for immunoblot analyses) were from Invitrogen (Carlsbad, CA, USA) or Santa Cruz Biotechnology (Santa Cruz, CA, USA).

### 2.4. Transfection and RNA Interference

Transfection was performed using Metafectene Pro from Biontex (Martinsried, Germany) according to the protocol provided by the manufacturer or using Amaxa Cell Line Nucleofector Kit V (Lonza, Basel, Switzerland) as previously described [26]. Cells were then analyzed after 24 h of transfection. Transfection of cells with small interfering RNAs (siRNAs) was performed using Oligofectamine from Life Technologies (Carlsbad, CA, USA) following the manufacturer’s instructions. siRNA transfection has been conducted for 72 h, then cells were replated and left for 48h before lysis. siRNAs were purchased from MWG-Biotech (Ebersberg, Germany). RAB7A siRNA efficiency in silencing was reported previously [24]. For RAB7A siRNA 1, sense sequence 5′-GGAUGACCUCUAGGAAGAATT-3′ and antisense sequence 5′-UUCUUCCUAGAGGUCAUCCTT-3′, for RAB7A siRNA 2, sense sequence 5′-GAACACACGUAGGCCUUCATT-3′ and antisense sequence 5′-UGAAGGCCUACGUGUGUUCTT-3′. Control RNA was used as a negative control: sense sequence is 5′-ACUUCGAGCGUGCAUGGCUTT-3′ while antisense sequence is 5′-AGCCAUGCACGCUCGAAGUTT-3′.

### 2.5. Co-Immunoprecipitation

For co-immunoprecipitation experiments, cells were lysed in lysis buffer (0.5% NP-40, 25 mM Tris, pH 7.5, and 100 mM NaCl supplemented with protease inhibitor mixture) and immunoprecipitation was performed using anti-HA resin (Ezview Red Anti-HA Affinity gel, Sigma-Aldrich), following the protocol provided by the manufacturer. Briefly, cell lysates were incubated at 4 °C with 25 µL of resin. After 1 h on a rotating wheel, immunoprecipitates were washed three times with washing buffer (0.1% NP-40, 25 mM Tris, pH 7.5, and 150 mM NaCl supplemented with a protease inhibitor mixture) and once with 10 mM Tris, pH 7.5, as previously described [27]. Finally, samples were subjected to SDS-PAGE and Western blot analyses.

### 2.6. Pull-Down Experiments

Pull-down experiments were performed as previously described [9]. Briefly, 20 µg of His (Histidine)-tagged RAB7A proteins expressed and purified from Escherichia Coli BL21 were bound to Ni-NTA (Nickel-Nitrilotriacetic Acid) resin and then incubated for 1 h at 4 °C with total extracts of HeLa cells overexpressing HA-tagged vimentin. After incubation, samples were subjected to SDS-PAGE and Western blot analysis.

### 2.7. Western Blotting

Cells were lysed with RIPA (Radioimmunoprecipitation Assay) buffer (50mM Tris-HCl, pH 8.0, with 150 mM sodium chloride, 1.0% Igepal CA-630 (NP-40), 0.5% sodium deoxycholate, and 0.1% sodium dodecyl sulfate) plus protease inhibitor cocktail (Roche, Mannheim Germany) and phosphatase inhibitors (phosphatase inhibitor cocktail 1 from Sigma-Aldrich). Lysates were loaded onto SDS-PAGE and separated proteins were transferred onto polyvinylidene fluoride (PVDF) membrane from Millipore (Billerica, MA, USA) and probed with the primary antibody diluted in 2% milk in PBS (Phosphate-Buffered Saline) followed by HRP-conjugated secondary antibody, as previously described [23]. Bands were visualized using Western blot Luminol Reagent (Santa Cruz), WesternBright ECL kit (Advansta, Menlo Park, CA, USA), Clarity (BioRad, Hercules, CA, USA), or Clarity Max (BioRad, Hercules, CA, USA), and signals were captured on a film or using Bio-Rad ChemiDoc MP Imaging Systems (Hercules, CA, USA).

### 2.8. Gelatin Zymography

HeLa cells were seeded, transfected with control RNA or RAB7A siRNA and the day after the replating, cells were cultured in serum-free medium for 48 h. Conditioned media were collected from cells, samples were concentrated using Amicon^®^ Ultra (Merck Millipore), quantified by Bradford assay and gelatin zymography was performed as previously described [28]. Briefly, after electrophoresis, gel was washed twice (30 min each at room temperature) in 50 mM Tris-HCl pH 7.5 containing 2.5% Triton X-100, 5 mM CaCl_2_, 1 µM ZnCl_2_ to remove SDS and this step was followed by three more washes in distilled water. After washing, the gel was incubated at 37 °C for 24 h in activation buffer containing 1% Triton X-100, 50 mM Tris-HCl pH 7.5, 5 mM CaCl_2_, and 1 µM ZnCl_2_, and then was stained with 0.5% Coomassie Blue R-250 (Sigma-Aldrich, St. Louis, MI, USA) and destained in 40% Methanol and 10% acetic acid.

### 2.9. Immunofluorescence and Confocal Microscopy

HeLa cells were fixed for 20 min in 3% paraformaldehyde and then permeabilized with 0.1% Triton X-100 in PBS. Subsequently, samples were quenched in NH_4_Cl for 10 min and then incubated at room temperature with primary and secondary antibodies diluted in 0.1% saponin in PBS. After several washes, coverslips were mounted with mowiol and viewed with Zeiss LSM 700 confocal microscope.

### 2.10. In-Silico Modeling

In order to model the 3D structure of the involved proteins, we used Modeller 9.24 [29] (via Chimera [30]) and I-Tasser [31]. Modeller was used to model RAB7A and the vimentin fragments, while I-Tasser was used to obtain the whole model of vimentin. This second server was chosen because structural and filamentous proteins, such as vimentin, are not characterized as a whole structure but often as a series of fragments. On the other hand, a homology-based approach is sufficient to obtain a high-quality model for RAB7A. So, for vimentin, which shows an uncharacterized globular structure at the head and tail domains, we used I-Tasser that combines ab-initio a homology-based method to model a protein. The other models used in this study were downloaded from the PDB (RCSB) database (https://www.rcsb.org/ accessed on 1 April 2021). To perform docking simulation two online servers PatchDock [32] and GrammX [33] were used. For both software, the energy calculation and complex refinement were performed using FireDock [34,35]. The RAB7A mutated structures were generated using Dynamut [36] which also analyzes the effects of the mutations on the stability of the folding. To compare RAB7A wild type and RAB7A^Y183H^ we used a dynamic approach (docking-based). To estimate the dynamic feature of the protein we used CABSflex server using as input the wild type model, Y183H and Y183F models. The two-sequences alignments were obtained using LALIGN [37]. Chimera was used to analyze the results and to acquire the images [30].

### 2.11. Luciferase Assays

HeLa cells were transfected with 5 ng of the NF-kB luciferase reporter vector and 1 μg of different expression vectors. Twenty-four hours after transfection, cells were lysed in Passive Lysis Buffer supplied by Promega (Madison, WI, USA) and luciferase activity in the cellular lysates was assessed on a Glomax 20/20 luminometer (Promega) using the Luciferase Assay System (Promega). Results were normalized for transfection efficiency by using a GFP coding vector (1 μg). All luciferase results represent the average ±S.D. of three independent experiments. All samples were read in triplicate.

### 2.12. Quantification and Statistical Analysis

Protein amounts were quantified by densitometry using the ImageJ software. Experiments were performed at least in triplicate and error bars represent standard error of the mean (S.E.M.). Data were statistically analyzed using Student’s *t*-test (GraphPad Prism4 software) (* *p* < 0.05, ** *p* < 0.01, and *** *p* < 0.001).

## 3. Results

### 3.1. RAB7A Interacts with the Coil 1 Domain of Vimentin

**Figure 1:**
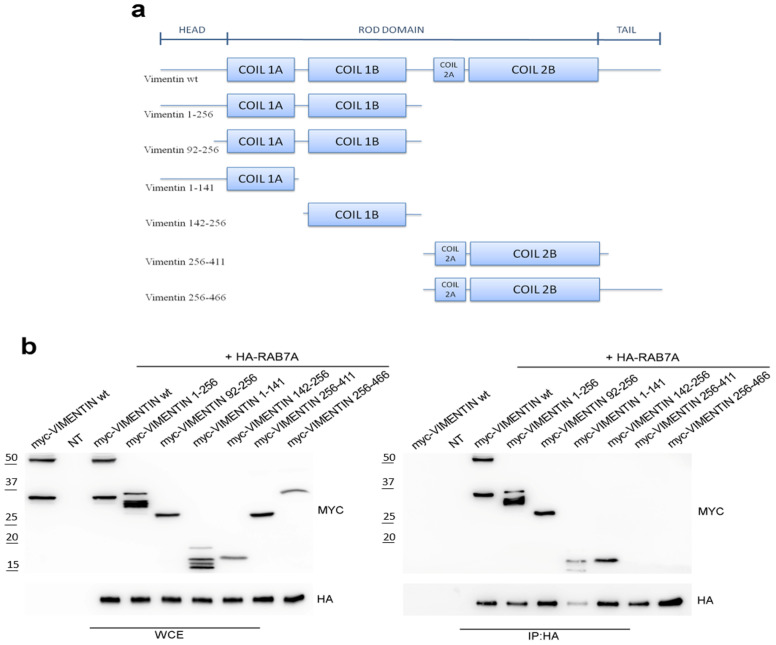
RAB7A interacts with the coil1 domain of vimentin. (**a**) scheme representing the structure of wild type vimentin and its deletion mutants constructed for testing the interaction with RAB7A. (**b**) hemagglutinin (HA)-tagged RAB7A and wild type or mutated myc-tagged vimentin proteins were expressed in HeLa cells as indicated. Immunoprecipitation (IP) has been performed using an anti-HA resin. Immunoprecipitates were subjected to Western blot analysis using anti-myc and anti-HA antibodies. Expression of the proteins of interest in the lysate before performing co-IP has been tested (Whole-Cell extract (WCE)).

We have previously discovered the interaction between RAB7A and vimentin by a two-hybrid screening and confirmed it with different techniques, including co-immunoprecipitation in HeLa cells [9]. However, the interaction domains of the two proteins had not been identified. In order to investigate the domains of vimentin interacting with RAB7A, vimentin deletion mutants were constructed and tested for interaction by performing co-immunoprecipitation. The deletion mutants of vimentin used are listed in Figure 1a.

Lysates of HeLa cells expressing HA-tagged RAB7A wild type and myc-tagged wild type or mutated vimentin were subjected to co-immunoprecipitation using anti-HA monoclonal antibody covalently attached to cross-linked agarose beads. Samples were run on SDS-PAGE and subjected to Western blot analysis. Interestingly, we found that RAB7A interacts with vimentin 1-256 and vimentin 92-256 (containing coil1 domain). As the coil1 domain is composed of the coil1A and coil1B domains, we evaluated the interaction of vimentin 1-141 (encoding head and coil1A domains) and vimentin 142-256 (encoding coil 1B) with RAB7A, demonstrating that RAB7A also interacts with these fragments but it does not interact with vimentin 256-411 and vimentin 256-466 (containing coil2 domain) fragments (Figure 1b). These data indicate that the vimentin domain interacting with RAB7A is comprised within aa 92 and 256. Sequencing of one of the positive clones isolated from the previously performed RAB7A two-hybrid interaction screening [9] revealed that this clone encoded the aa 208-466 of vimentin, thus confirming the interaction between aa 208 and 256. Therefore, not only vimentin coil1B domain (aa 142-256) was able to interact with RAB7A, in line with the two-hybrid screening results, but also the vimentin deletion mutant containing head and coil1A domains (aa 1-141), demonstrating that there is more than one domain of vimentin interacting with RAB7A.

**Figure 2:**
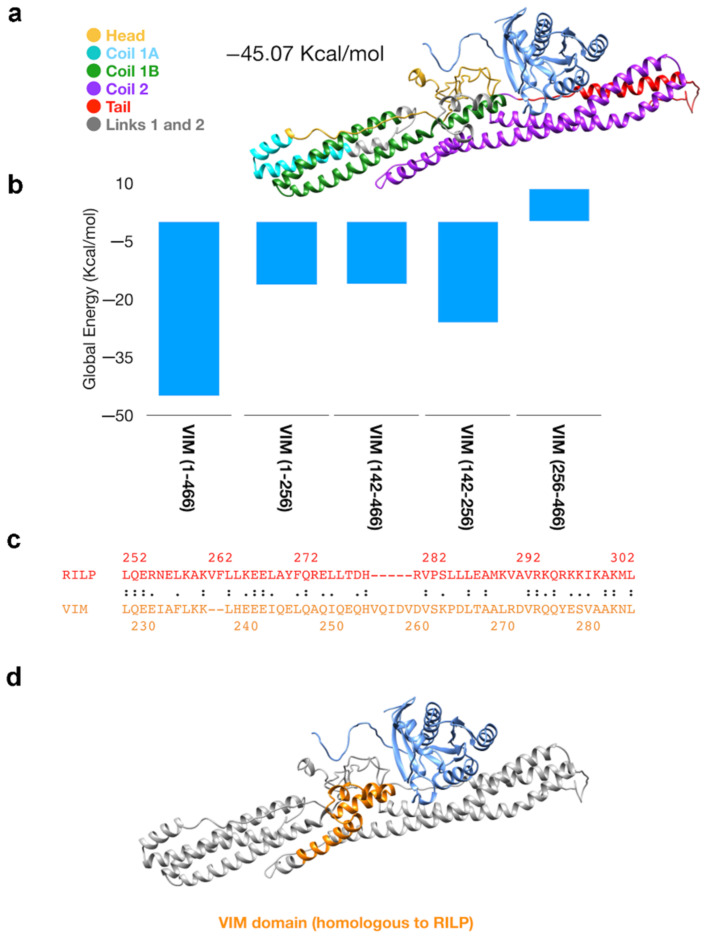
Docking results and 3D model of vimentin-RAB7A complex. (**a**) 3D structure of the best result obtained performing Gramm-X followed by FireDock simulations. We used the vimentin model as the receptor and RAB7A model as the ligand (cornflower blue). The domains of vimentin are marked in different colors as indicated. (**b**) results obtained by Patchdock and FireDock simulation using as receptor the truncated models of the vimentin. (**c**) homology between the sequence of Rab interacting lysosomal protein (RILP) (red) and the sequence of vimentin (orange) calculated by LALIGN server. (**d**) 3D representation of vimentin-RAB7A complex (showed also in panel A) with the region homologous to RILP marked in orange.

To analyze structurally this interaction, we performed docking simulations using Gramm-X (for the whole model) or PatchDock (for the fragments), as detailed in Materials and Methods. This model confirmed the co-immunoprecipitation data as it revealed the interaction of RAB7A with the vimentin coil 1B domain but also with the vimentin head domain, while no interaction was detected with coil2 or tail domains (Figure 2a,b, Tables S1 and S2).

Interestingly, a homology search revealed that a string of about fifty amino acid in the coil1B domain of vimentin displays 30% of identity and 52% similarity with the RAB7A-interacting domain of Rab interacting lysosomal protein (RILP), described previously [38]. Moreover, the first 30 amino acids of this string display 30% of identity and 70% of similarity with the RILP domain (Figure 2c). In Figure 2d, the vimentin domain similar to the RILP domain interacting with RAB7A is depicted.

Thus, we demonstrated that vimentin contains at least two domains interacting with RAB7A and one of them shares similarity with the RAB7A-interacting domain of RILP.

### 3.2. Mutation of Amino Acid 183 of RAB7A Affects the Interaction with Vimentin

**Figure 3:**
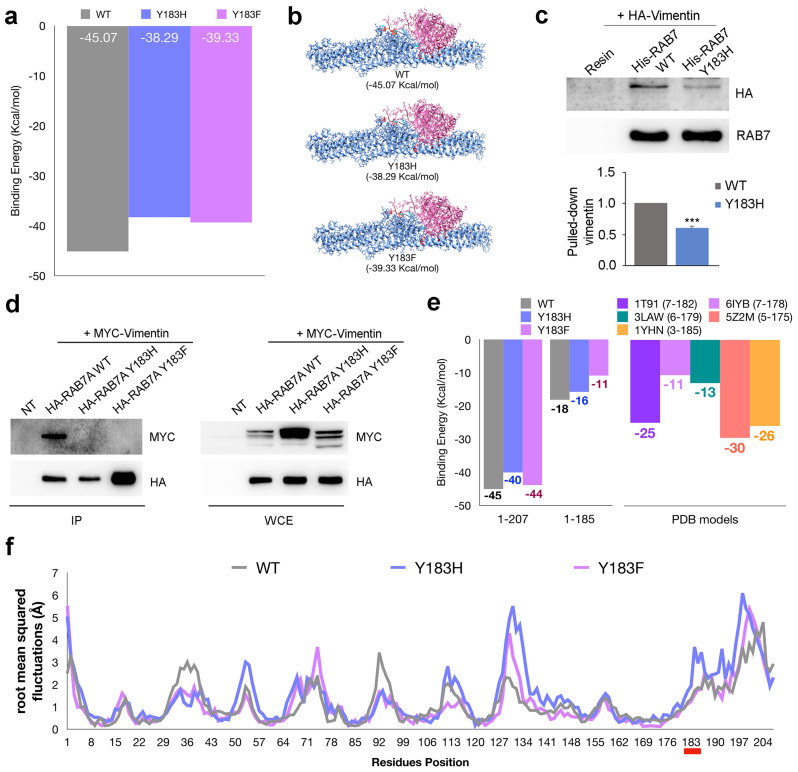
Interaction between vimentin and the RAB7AY183 mutant proteins. (**a**) results of docking between vimentin and wild type or mutated RAB7A. (**b**) 3D structure of the complexes between vimentin and RAB7A wild type, Y183H or Y183F obtained by Gramm-X docking simulations. (**c**) bacterially expressed and purified His-tagged RAB7A wild type and Y183H were incubated with total extracts of HeLa cells overexpressing HA-vimentin and pulled down with Ni-NTA resin. Proteins were subjected to Western blot analysis using anti-HA and anti-RAB7A antibodies. Quantification of pulled-down vimentin is shown. Data represent the mean ± s.e.m. of three independent experiments. *** = *p* < 0.001. (**d**) HA-tagged RAB7A wild type, Y183H or Y183F and myc-tagged vimentin wild type proteins were expressed in HeLa cells. IP has been performed using anti-HA resin. Immunoprecipitates were subjected to Western blot analysis using anti-myc and anti-HA antibodies. Protein expression in the lysates before performing co-IP has been checked (WCE). (**e**) Left: results of simulations of Gramm-X docking using RAB7A complete models (1-207) and RAB7A truncated models (1-185); Right: results of PatchDock simulations using as ligands different models of RAB7A downloaded from the PDB (RSCB) database. In the legend start and stop residues for each model are shown. (**f**) fluctuation plots obtained performing CABSflex analysis on RAB7A wild type and Y183H and Y183F mutant models.

To further analyze the interaction between RAB7A and vimentin, we investigated the domains of RAB7A interacting with vimentin using Chimera to prepare the input file while GrammX and FireDock were used as simulation tools. This in silico analysis showed that RAB7A interacts with vimentin both with the globular domain and with its C-terminal domain. To establish if the Y183 amino acid, which can be phosphorylated, is involved in the interaction, we looked at the RAB7A^Y183H^ and RAB7A^Y183F^ mutants discovering that they show a higher energy value for both Y183H and Y183F models (an increase of 15% for RAB7A^Y183H^ and 13% for RAB7A^Y183F^) if compared with the wild-type protein (Figure 3a, Table S3).

This difference is related to the rearrangement of lateral chains of the residues rather than to a change of the interaction site, as shown by structural analysis of the docking complex performed by Chimera (Figure 3b, Table S4).

To confirm the data experimentally we produced the RAB7A^Y183H^ mutant in bacteria and we analyzed its ability to pull down vimentin from total extracts of HeLa cells compared to RAB7A wild type. Interestingly, the RAB7A^Y183H^ mutant was less efficient in pulling down vimentin from total extracts compared to RAB7A wild type (Figure 3c).

To test if RAB7A phosphorylation is required for the interaction with vimentin, we performed a co-immunoprecipitation assay in HeLa cells expressing myc-tagged vimentin and HA-tagged RAB7A wild type, Y183H or Y183F. Interestingly, both RAB7A mutants were not able to interact with vimentin suggesting that dephosphorylated RAB7A cannot interact with its effector (Figure 3d). As shown in the table of panel a (Figure 3a) most of the bonds, revealed by structural analysis, map on the C-terminal domain of RAB7A. To clarify the effect of the Y183H and the Y183F mutations on the C-terminal domain we manually modified the wild type, RAB7A^Y183H^ and RAB7A^Y183F^ docking complexes, to obtain truncated models. The results of the energy estimation performed by FireDock (Figure 3e left) demonstrate the importance of the C-terminal domain for the interaction between RAB7A and vimentin. This effect was confirmed using native RAB7A models downloaded by RCSB (Figure 3e right, Table S5). Finally, RAB7A^Y183H^ and RAB7A^Y183F^ showed a different dynamic feature if compared with RAB7A^WT^ (Figure 3f, Table S6). In particular, at position 183 the fluctuation plot shows an increase of the flexibility in the RAB7A^Y183H^ mutant model. On the other hand, RAB7A^Y183F^ shows flexibility comparable with wild type model around at position 183, while an increase of flexibility was observed in another region (residues 71–78). Finally, for both RAB7A^Y183H^ and RAB7A^Y183F^ a flexibility increase was observed in the 127 to 134 region and at the C-terminal domain of RAB7A, while an opposite effect was shown in the domains around residues 92 and 36.

### 3.3. Modulation of RAB7A Expression Affects AKT and PAK1 but Not ROCK2 and PKA Kinases

**Figure 4:**
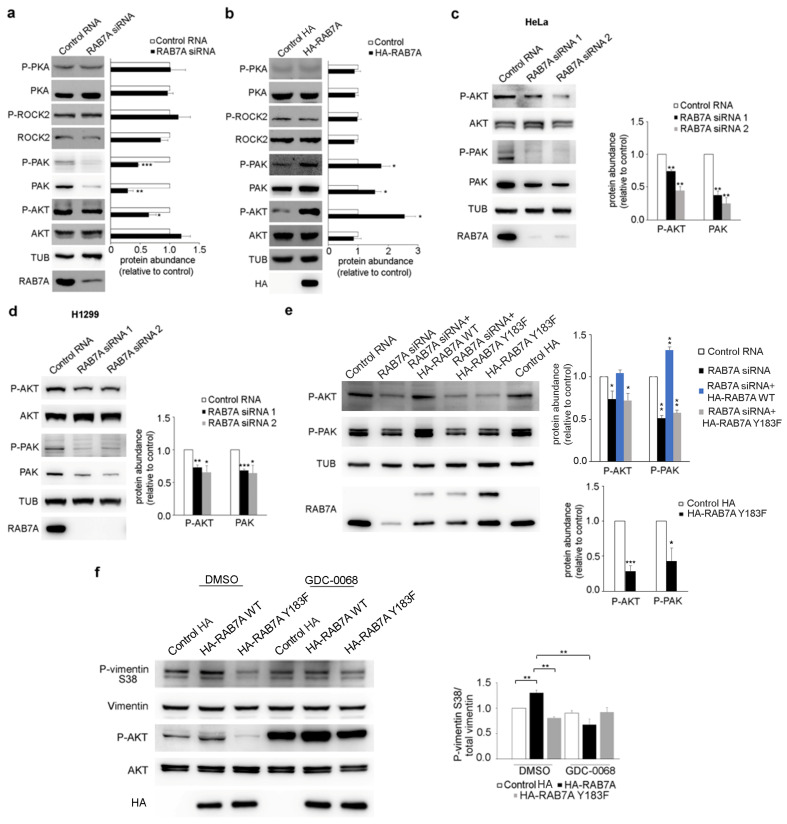
RAB7A regulates AKT kinase activity, PAK1 abundance, and vimentin phosphorylation. (**a**) HeLa cells were transfected with either control RNA or RAB7A siRNA and then lysed after 5 days or (**b**) transfected with plasmids encoding either HA or HA-RAB7A and lysed after 24 h. Lysates were subjected to Western blot analysis using the indicated antibodies. (**c**) HeLa or (**d**) H1299 cells were treated with control RNA or with RAB7A siRNAs and lysed after 5 days. Lysates were subjected to Western blot analysis using the indicated antibodies. Quantifications of total PAK kinase and phosphorylated AKT kinase are shown. (**e**) HeLa cells were treated with control RNA or with RAB7A siRNA or transfected with plasmids coding for HA or HA-RAB7A^Y183F^. RAB7A-silenced cells were also transfected with plasmids encoding HA-RAB7A WT or Y183F to rescue RAB7A function. Lysates were subjected to Western blot analysis using the indicated antibodies. Quantification of phosphorylated AKT and PAK1 kinases was shown. (**f**) HeLa cells were transfected with plasmids encoding either HA, HA-RAB7A^WT^, or HA-RAB7A^Y183F^. 24 h after transfection cells were incubated with the AKT inhibitor GDC-0068 1 µM or DMSO for 24 h and then lysed. Lysates were subjected to Western blot analysis using the indicated antibodies. Quantification of P-vimentin Ser38 is shown. All data represent the mean ± s.e.m. of at least three independent experiments. * = *p* < 0.05; ** = *p* < 0.01; *** = *p* < 0.001.

Given the regulation of RAB7A on vimentin phosphorylation state, we have analyzed the activation state of AKT, PKA, PAK1, and ROCK2 upon RAB7A silencing in HeLa cells by evaluating the abundance of these kinases and of their activated forms through Western blot analysis (Figure 4a).

We observed that depletion of RAB7A did not alter PKA and ROCK2 abundance or activity (Figure 4a). However, RAB7A silencing significantly reduced the amount of activated AKT but not its total abundance in HeLa cells (Figure 4a), in line with previous results obtained in A431 cells during anoikis (matrix-detachment triggered apoptosis) or EGF (Epidermal Growth Factor) stimulation assays [39]. In particular, we detected a reduction of activated AKT of about 30% upon RAB7A silencing (Figure 4a). Furthermore, PAK1 was affected by the depletion of RAB7A; in fact, the abundance of this kinase and of its phosphorylated form was reduced by about 70% when RAB7A was knocked down (Figure 4a).

Having demonstrated that RAB7A depletion influences AKT and PAK1 kinases and given the fact that over-expression of RAB7A determined an increase in the amount of Ser38 and Ser55 phosphorylated vimentin [9], we analyzed the activation state of AKT, PKA, PAK1, and ROCK2 after over-expression of RAB7A (Figure 4b). We observed that over-expression of RAB7A did not alter PKA and ROCK2 abundance or activity (Figure 4b). Interestingly, however, RAB7A over-expression caused a strong increase of activated AKT but did not change its total abundance (Figure 4b). In particular, we observed a 2.5-fold increase of activated AKT after expression of HA-RAB7A compared to control cells transfected with the plasmid encoding only HA (Figure 4b). Moreover, upon over-expression of RAB7A, the total amount of PAK increased and consequently also its phosphorylated form (Figure 4b).

To further validate our data, we treated HeLa cells with another RAB7A siRNA and we evaluated PAK1 and AKT abundance and activation. As expected, using both siRNA, we obtained similar results (Figure 4c). Indeed, we found a reduction of active AKT and a reduction of total and active PAK1 upon RAB7A silencing with both siRNAs (Figure 4c). Furthermore, we also silenced RAB7A in H1299 cells using both siRNAs demonstrating that, also in these cells, PAK1 abundance and AKT phosphorylation were affected by RAB7A expression (Figure 4d). As we had validated our siRNAs in two different cell lines and demonstrated that our results were not due to off-target effects, we used RAB7A siRNA 1 in HeLa cells hereafter.

Having demonstrated that RAB7A silencing affects AKT and PAK1, we transfected HeLa cells with plasmids coding for HA or HA-RAB7A^Y183F^, to test if the expression of RAB7A mutant protein also diminished AKT activation and phosphorylated PAK1. Interestingly, similarly to RAB7 silencing, the expression of RAB7A^Y183F^ reduced P-AKT (Phosphorylated-AKT) and P-PAK1 (Phosphorylated-PAK1) abundance in HeLa cells (Figure 4e). Moreover, we transfected HeLa cells silenced for RAB7A with plasmids encoding RAB7A WT or Y183F, to evaluate if the expression of these proteins was able to rescue the effects caused by silencing. RAB7A^WT^ re-expression caused an increase in P-AKT and P-PAK1 demonstrating that the decrease of both these proteins is directly related to RAB7A silencing and it is not a consequence of off-target effects of the siRNA (Figure 4e). On the contrary, the mutant protein RAB7A^Y183F^ was not able to rescue RAB7A function after silencing since its expression failed to increase P-AKT and P-PAK1 abundance (Figure 4e).

These results highlight that modulation of RAB7A expression in different cell lines affects PAK1 and AKT kinases.

### 3.4. RAB7A Regulates Vimentin Phosphorylation at Ser38 Modulating AKT Activity

It has been demonstrated that RAB7A overexpression causes increased phosphorylation of vimentin at Ser38 and that this residue is phosphorylated by AKT, enhancing cellular motility and invasion [9,11]. The fact that RAB7A is able to regulate AKT activity prompted us to hypothesize that the increased vimentin phosphorylation at Ser38 observed in RAB7A overexpressing cells could be due to AKT activity modulated by RAB7A. To demonstrate this, we decided to treat HeLa cells with the AKT inhibitor GDC-0068. This chemical is an ATP-competitive pan-Akt inhibitor that induces a dose-dependent increase in Akt phosphorylation [40]. Consistently with this, as shown in Figure 4f, we observed a strong inhibition of AKT due to the treatment with GDC-0068 and increased vimentin phosphorylation at Ser38 in RAB7A overexpressing cells, confirming data obtained in our previous work [9]. Furthermore, we demonstrated that the RAB7A^Y183F^ mutant caused a statistically significant reduction of vimentin phosphorylation at Ser38, accordingly to the decrease of AKT activation upon RAB7A^Y183F^ expression. Moreover, we demonstrated that enhanced vimentin phosphorylation at Ser38 was lost in RAB7A overexpressing cells following the treatment with GDC-0068 and this result was statistically significant compared to RAB7A overexpressing cells treated only with DMSO (Figure 4f).

These data demonstrate that RAB7A regulates vimentin phosphorylation at Ser38 through regulation of AKT activity.

### 3.5. RAB7A Regulates Beta-Catenin and Caspase 9 Expression

**Figure 5:**
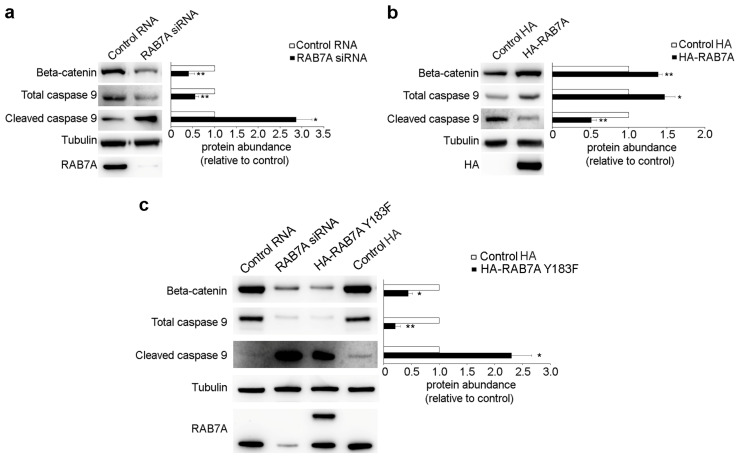
Modulation of RAB7A expression acts on beta-catenin and caspase 9 expression. (**a**) HeLa cells were transfected with either control RNA or RAB7A siRNA, replated 72 h after transfection and then lysed after 48 h. (**b**) HeLa cells were transfected with plasmids encoding either HA or HA-RAB7A and lysed after 24 h. (**c**) HeLa cells were treated with either control RNA or RAB7A siRNA and lysed after 5 days or were transfected with plasmids encoding HA or HA-RAB7A^Y183F^ and lysed after 24 h. Lysates were subjected to Western blot analysis using anti-beta catenin, anti-caspase 9, anti-cleaved caspase 9, anti-tubulin, anti-HA, and anti-RAB7A antibodies. Quantifications of beta-catenin, caspase 9, and cleaved caspase 9 are shown. All data represent the mean ± s.e.m. of at least three independent experiments. * = *p* < 0.05; ** = *p* < 0.01.

AKT and PAK kinases are key regulators of several cellular events such as proliferation, apoptosis, and survival [41,42]. Since we demonstrated that RAB7A regulates AKT activity and PAK1 abundance, consequently influencing its activity, we evaluated downstream effectors of these kinases upon RAB7A silencing or over-expression. Among them, we analyzed the expression of beta-catenin and we found that RAB7A silencing caused a reduction of about 60% of its abundance (Figure 5a).

Moreover, beta-catenin expression was increased by about 50% in cells overexpressing RAB7A (Figure 5b).

AKT and PAK are both implicated in apoptosis since activation of these kinases prevents the processing of procaspase-9, enhancing survival [43,44]. Thus, we also investigated if modulation of RAB7A expression affects caspase 9 cleavage and we found a reduction in caspase 9 total abundance accompanied by increased cleavage of this caspase upon RAB7A silencing (Figure 5a). Consistently, we observed increased caspase 9 abundance and reduced cleavage in RAB7A overexpressing cells (Figure 5b). Moreover, to test the effects of the RAB7A^Y183F^ mutant protein, we transfected HeLa cells with plasmids encoding HA or HA-RAB7A^Y183F^ and we analyzed the abundance of beta-catenin and of the total and cleaved caspase 9. As shown in Figure 5c, the presence of the RAB7A mutant protein caused reduced expression of beta-catenin and procaspase-9 and increased caspase 9 cleavage, similarly to the effects produced by RAB7A silencing (Figure 5c).

### 3.6. RAB7A Affects NF-kB Expression and Localization

**Figure 6:**
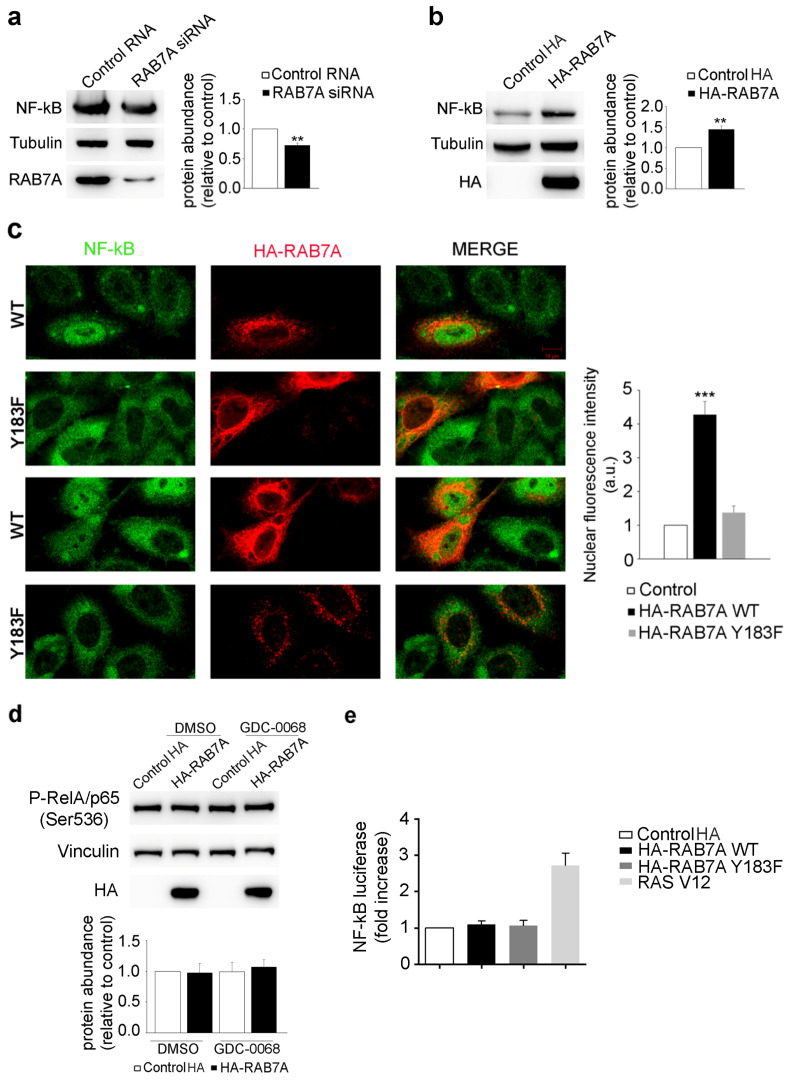
RAB7A affects NF-kB expression and localization. (**a**) HeLa cells were transfected with either control RNA or RAB7A siRNA, replated 72 h after transfection and then lysed after 48 h. (**b**) HeLa cells were transfected with plasmids encoding either HA or HA-RAB7A and lysed after 24 h. Lysates were subjected to Western blot analysis using anti-NF-kB, anti-tubulin, anti-RAB7A and anti-HA antibodies. The quantification of NF-kB is shown. Data represent the mean ± s.e.m. of at least three independent experiments. (**c**) HeLa cells were transfected with a plasmid coding for HA-RAB7A^WT^ or HA-RAB7A^Y183F^ and, 24 h after transfection, were analyzed by immunofluorescence using anti-HA (red) and anti-NF-kB (green) antibodies. Scale bar = 10 µM. NF-kB nuclear fluorescence intensities of at least 50 cells per sample were measured using ImageJ. Data represent the mean ± s.e.m. of three independent experiments. Statistical analyses were performed using Student’s *t* test with control cells as referring sample. (**d**) HeLa cells were transfected with plasmids encoding either HA, HA-RAB7A^WT^ or HA-RAB7A^Y183F^. Then, 24 h after transfection cells were treated with AKT inhibitor GDC-0068 1 µM or DMSO for 24 h and then lysed. Lysates were subjected to Western blot analysis using anti-P-p65/RelA (Ser536), anti-vinculin and anti-HA antibodies. (**e**) HeLa cells were transfected with the NF-kB luciferase reporter vector and with different expression vectors. Twenty-four hours after transfection, cells were lysed and the luciferase activity was measured in cell extracts. Data are represented as fold induction of the normalized luciferase activity with respect to control cells transfected with GFP. All luciferase results represent the average ± S.D. of three independent experiments. All samples were read in triplicate. ** = *p* < 0.01; *** = *p* < 0.001.

NF-kB, a transcription factor regulating inflammation, transformation, proliferation, angiogenesis, invasion, metastasis, and chemoresistance in cancer, is modulated by AKT and PAK1 [45,46,47]. Considering the role of RAB7A on these two kinases, we evaluated if NF-kB expression was affected by modulating RAB7A expression. We demonstrated that RAB7A silencing leads to a reduction in NF-kB total abundance of about 30% (Figure 6a) while RAB7A over-expression causes its increased expression of around 50% (Figure 6b). In order to determine if RAB7A affects NF-kB cellular localization, we performed an immunofluorescence assay demonstrating that, in RAB7A overexpressing cells, NF-kB is more present in the nuclei compared to non-transfected cells, thus possibly increasing its activity as a transcription factor. In contrast, in RAB7A^Y183F^ expressing cells, NF-kB localization is cytosolic, similarly to what happens in non-transfected cells (Figure 6c).

NF-kB can be phosphorylated at serine 536 in the transactivation domain (TAD) by several kinases and this modification leads to increased transactivation [48]. Among these kinases, IκB (Inhibitor of NF-kB) kinase α (IKKα) is phosphorylated by AKT and, once activated by AKT, it phosphorylates the IκB protein and also p65/RelA, inducing increased activation of NF-kB [46]. To test if RAB7A overexpression affects NF-kB phosphorylation, we performed a Western blot analysis using an antibody that recognized p65/RelA phosphorylated at serine 536. As shown in Figure 6d, NF-kB phosphorylation does not change in RAB7A overexpressing cells, suggesting that RAB7A does not affect NF-kB activation. To further investigate this issue, we performed a luciferase assay, which revealed that neither RAB7A^WT^ nor RAB7A^Y183F^ could stimulate NF-kB-dependent transcription, a result instead obtained with the positive control Ras V12 (Figure 6e).

Altogether these data indicate that RAB7A affects NF-kB expression and its nuclear localization while it does not influence NF-kB activation.

### 3.7. RAB7A Regulates Cofilin-1 Abundance and MMP2 Activity

**Figure 7:**
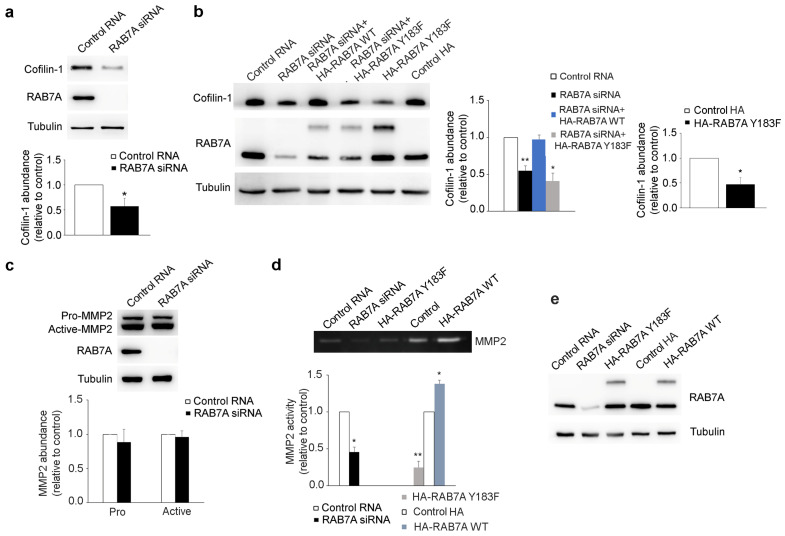
RAB7A regulates cofilin-1 abundance and matrix metalloproteinase 2 (MMP2) activity. (**a**) HeLa cells were transfected with either control RNA or RAB7A siRNA, replated 72 h after transfection and then lysed after 48 h. Lysates were subjected to Western blot analysis using the indicated antibodies. Quantification of cofilin-1 abundance is shown. (**b**) HeLa cells were treated with either control RNA or RAB7A siRNA or transfected with plasmids coding for HA or HA-RAB7A^Y183F^. HeLa cells silenced for RAB7A were also transfected with plasmids encoding HA-RAB7A^WT^ or Y183F to rescue RAB7A functions. Lysates were subjected to Western blot analysis using the indicated antibodies. Quantification of cofilin-1 abundance is shown. (**c**) HeLa cells were transfected with either control RNA or RAB7A siRNA, replated 72 h after transfection and then lysed after 48 h. Lysates were subjected to Western blot analysis using anti-MMP2, anti-tubulin, and anti-RAB7A antibodies. Quantifications of pro-MMP2 and active-MMP2 abundance are shown. (**d**) gelatin zymography was performed using conditioned medium of HeLa cells treated with either control RNA or RAB7A siRNA or transfected with plasmids encoding HA, HA-RAB7A^WT^, or HA-RAB7A^Y183F^. Quantification of MMP2 activity is shown. (**e**) HeLa cells, treated with either control RNA or RAB7A siRNA or transfected with plasmids encoding HA, HA-RAB7A^WT^, or HA-RAB7A^Y183F^ and whose conditioned medium was used for gelatin zymography, were lysed and lysates were subjected to Western blot analysis using anti-tubulin and anti-RAB7A antibodies. All data represent the mean ± s.e.m. of three independent experiments. * = *p* < 0.05; ** = *p* < 0.01.

It was previously demonstrated that RAB7A has a role in cell migration [23]. Cofilin-1 is an actin-binding protein that regulates actin filament dynamics and it is required for cellular migration [49]. Cofilin-1 is active in its dephosphorylated form and it was previously shown that PI3K (Phosphatidylinositol 3-Kinase) and its downstream effector AKT activate the Slingshot phosphatase, increasing the rate of active cofilin-1 [50]. Considering the role of RAB7A in cellular migration and in regulating AKT activity, we decided to evaluate cofilin-1 expression in HeLa cells upon RAB7A silencing and we demonstrated that RAB7A depletion reduces cofilin-1 abundance by about 50% compared to control cells (Figure 7a).

To test the effect of RAB7A^Y183F^ expression on cofilin-1 abundance, we transfected HeLa cells with plasmids coding for HA or HA-RAB7A^Y183F^ mutant protein and, through Western blot analysis, we demonstrated that the mutant protein diminished cofilin-1 abundance (Figure 7b). Moreover, we transfected HeLa cells silenced for RAB7A with plasmids encoding HA-RAB7A^WT^ or Y183F to evaluate if these proteins can rescue RAB7A function after silencing. As shown in Figure 7b, while the re-expression of RAB7A^WT^ increased cofilin-1 abundance, the mutant protein could not rescue RAB7A function.

A recent work showed that cofilin-1 depletion suppresses cell migration and decreases MMP2 activity [51]. Having demonstrated that RAB7A silencing affects cofilin-1 abundance, we evaluated MMP2 expression and activity in HeLa cells upon RAB7A silencing. We did not find any differences in MMP2 protein levels between cells transfected with control RNA or RAB7A siRNA (Figure 7c), but when we analyzed MMP2 activity by gelatin zymography, we observed reduced activity of this enzyme in RAB7A-depleted cells (Figure 7d,e). Furthermore, while the expression of HA-RAB7A^WT^ increased MMP2 activity, the opposite effect was caused by HA-RAB7A^Y183F^ which reduced MMP2 activity by about 75% compared to control cells (Figure 7d).

Altogether these results indicate that RAB7A regulates cell migration modulating cofilin-1 abundance and MMP2 activity.

## 4. Discussion

Vimentin is an intermediate filament protein whose assembly from monomers to filaments is regulated by phosphorylation [52]. We previously demonstrated that the small GTPase RAB7A interacts with vimentin and regulates its phosphorylation state and assembly influencing cell migration [9,23].

Here, we demonstrated that RAB7A interacts with coil1B domain of vimentin, a domain containing a stretch of amino acids sharing similarity with the RAB7A-binding domain present in RILP, and with the head but not with coil2 and tail domains. The central α-helical rod domain of the vimentin monomer is flanked by non-α-helical N- and C-terminal domains, constituting the head and tail domains, respectively [52]. In particular, the head domain folds back onto the coil1 domain of the rod region and its phosphorylation induces structural changes in adjacent dimer [53,54]. Therefore, RAB7A, interacting with the head and coil1B domains, could directly modulate vimentin assembly impacting the interactions between dimers and/or tetramers. Furthermore, we demonstrated that the amino acid 183, a tyrosine that has been previously demonstrated to be phosphorylated [55], is important for the interaction.

To understand how RAB7A could regulate the phosphorylation state of the vimentin head domain, we tested a number of kinases and established that silencing of RAB7A decreased AKT activity and PAK1 abundance and, consequently, its activity while no effect was detected on PKA and ROCK2. This is in line with previous works showing in A431 cells reduced amount of activated AKT upon RAB7A depletion during anoikis (matrix-detachment triggered apoptosis) or EGF stimulation assays [39] and in NCI H1299 cells decreased activation of RAC1, of which PAK1 is a major downstream effector [56]. As AKT and PAK1 are responsible for vimentin phosphorylation at Ser38 and Ser55 residues, respectively [11,13], lower activity of AKT and PAK1 after RAB7A silencing could explain the reduced Ser38 and Ser55 phosphorylation of vimentin upon RAB7A depletion detected previously [9]. Conversely, overexpression of RAB7A increases both Ser38 and Ser55 phosphorylated vimentin and vimentin abundance in the soluble pool [9]. In line with this, overexpression of RAB7A increased AKT activity and PAK1 abundance, thus influencing also PAK1 activity, while PKA and ROCK2 abundance and activity were not affected. In addition, the use of the AKT inhibitor GDC-0068 allowed us to demonstrate that RAB7A regulates vimentin phosphorylation at Ser38 modulating AKT activity. Moreover, we demonstrated that the expression of RAB7A^Y183F^ mutant diminished the amount of phosphorylated vimentin at Ser38, in agreement with the reduced activation of AKT caused by the presence of this mutant. Considering that the Y183F aminoacidic substitution caused an altered interaction between RAB7A and vimentin, these data suggest that the proper interaction between these two proteins is important for AKT activity.

**Figure 8:**
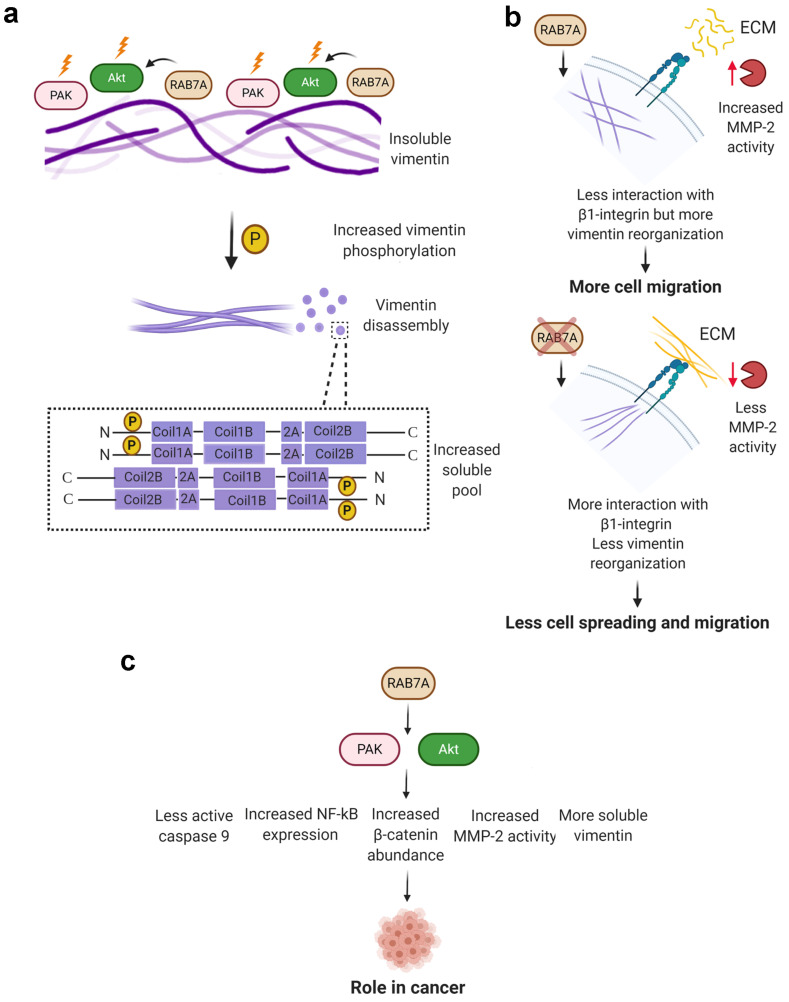
RAB7A regulation of vimentin assembly and of several AKT and PAK effectors. (**a**) RAB7A interacts with vimentin and activates AKT and PAK which in turn phosphorylate vimentin head domain. This increased phosphorylation leads to the disassembly of vimentin filaments in soluble tetramers made of two antiparallel dimers. (**b**) expression of RAB7A leads to vimentin filaments reorganization, increased MMP2 activity, and faster cell migration (top), whereas depletion of RAB7A determines alteration of vimentin filaments reorganization, reduced MMP2 activity, and slower cell migration (bottom). (**c**) schematic overview of RAB7A regulation on AKT and PAK kinases and their downstream effectors in cell migration, apoptosis, and cancer. Created with Biorender.com (accessed on 1 April 2021).

Recently, it has been proved that regulation of vimentin phosphorylation and assembly are important for the interaction between vimentin and β1-integrin and for cell adhesiveness on fibronectin substrate [22]. Indeed, co-expression of vimentin wild type and talin head domain increased adhesiveness of integrin α5β1 to fibronectin but not when vimentin was phosphorylated at Ser38 residue [22]. The phosphorylation state of vimentin is important in the short term, but the organization of vimentin filaments is of relevance in the long term as proved by the treatment with withaferin A (WFA), which induces perinuclear aggregation of vimentin filaments, phosphorylation of vimentin Ser38 and Ser55 residues and decreases the amount of soluble vimentin [22,57]. Differently from WFA, RAB7A silencing decreases the amount of soluble vimentin but decreases also phosphorylation of Ser38 and Ser55 residues of vimentin [9]. Our recent finding on the role of RAB7A in cell migration corroborates the data of Kim and colleagues [23]. In fact, RAB7A silencing decreases cell adhesion and spreading on fibronectin substrate by altering the activation state of β1-integrin, and affects vimentin filament reorganization during migration [23]. Thus, the lower amount of soluble vimentin and impaired reorganization of vimentin filaments induced by the lack of RAB7A, by misregulating AKT activity and PAK1 abundance, could result in a decrease of cell adhesion to fibronectin (Figure 8).

AKT and PAK kinases are also key regulators of proliferation, apoptosis and survival and they are overexpressed or hyperactivated in different types of cancers [41,42]. Modulation of RAB7A expression affects AKT and PAK1 downstream effectors. Beta-catenin is the major cellular effector of the Wingless and Int1 (Wnt) signaling, which causes proliferation of cancer cells as, upon Wnt activation, accumulated beta-catenin enters the nucleus and induces the expression of its target genes [58,59]. We found that RAB7A silencing reduces beta-catenin abundance, while RAB7A overexpression increases beta-catenin protein levels. In line with this, downregulation of PAK1 in colon cancer cells was demonstrated to reduce beta-catenin levels and cell proliferation [60]. Moreover, AKT phosphorylates beta-catenin that dissociates from cell-cell contacts and accumulates in the cytosol and in the nucleus, enhancing its transcriptional activity and promoting cancer cell invasion [61]. Thus, RAB7A could play a role in cancer cell proliferation and invasion regulating beta-catenin stability through AKT and PAK1. Another downstream effector of PAK1 and AKT is caspase 9, whose cleavage correlates with initiation of the apoptosis cascade [62]. RAB7A silencing increased caspase 9 cleavage while RAB7A overexpression caused a decrease in caspase 9 cleavage and an increase of pro-caspase 9 levels. AKT phosphorylates caspase 9 inhibiting its activity and thus apoptosis [44]. Furthermore, in breast epithelial MCF10A cells, expression of dominant-negative PAK1 increased caspase 9 cleavage while a PAK1 constitutively active mutant inhibited caspase 9 activation [43]. According to these results, RAB7A could regulate apoptosis acting on PAK1 and AKT.

We also evaluated the effects on NF-kB, a transcription factor constitutively active in most cancers, controlling proliferation, angiogenesis, invasion, metastasis, chemoresistance, and radioresistance [45]. Normally, NF-kB is sequestered in the cytosol by IκB that masks its nuclear localization signal [63]. RAB7A silencing reduced NF-kB abundance while RAB7A overexpression caused not only increased NF-kB protein levels but also nuclear translocation compared to control cells. Notably, phosphorylation of IκB causes IκB degradation and consequent NF-kB nuclear translocation, and IκB is an AKT substrate [46,64]. The role of PAK1 in NF-kB activation is less understood but it was demonstrated that PAK1 stimulates nuclear translocation of the p65 subunit of NF-kB in fibroblasts and macrophages although there is no evidence regarding IκB phosphorylation by PAK1 [65]. Altogether, these results suggest that RAB7A could regulate NF-kB nuclear translocation through activation of AKT and PAK1 (Figure 8). We also checked if RAB7A overexpression caused an increased activity of NF-kB as a transcription factor, following its augmented nuclear translocation. We found that RAB7A overexpression is not able to activate NF-kB, as demonstrated by luciferase assay and p65/RelA phosphorylation. Additionally, expression of the RAB7^Y183F^ mutant protein was not able to induce the translocation of NF-kB in the nucleus. These results are in line with evidence from the literature showing that NF-kB-dependent transcription is not regulated only by p65 nuclear localization, suggesting the existence of other factors required for NF-kB activation [66]. Accordingly, for instance, it has been shown that in GSK-3β (Glycogen Sinthase Kinase-3β) null cells, NF-kB function is reduced, although degradation of IκB and nuclear translocation of p65 were unaffected [67]. Thus, RAB7A regulates nuclear translocation of p65 but not its activity, suggesting that other factors are necessary for activation.

The rates of polymerization and depolymerization of actin cytoskeleton are determinant for cell motility and cofilin-1 is fundamental for actin dynamics [50,68]. We found that RAB7A-depleted cells show reduced cofilin-1 protein levels suggesting that RAB7A could affect cell migration also by regulating cofilin-1. In line with this, cofilin-1 silencing reduces colorectal cancer cell migration and invasion rates, as well as MMP2 activity [51]. Consistently, we observed decreased MMP2 activity, which correlates to AKT and PAK1. Indeed, in MKN45 gastric cancer cells, PAK1 silencing decreased mRNA levels and activity of MMP2 while PAK1 overexpression induced increased mRNA expression and increased activity of MMP2 leading to higher invasive properties [69]. Furthermore, in glioblastoma cell lines, it was demonstrated that Cyclosporin A affects migration and invasion by impairing PI3K/AKT pathway and the reduced cell motility correlates to diminished MMP2 gelatinolytic activity and to downregulated NF-kB activation [70]. MT1-MMP (Membrane Type 1-Matrix Metalloproteinase) is required for MMP2 activation, and RAB7A regulates endosomal trafficking and recycling of MT1-MMP together with VAMP7 (Vesicle Associated Membrane Protein), affecting migration and invasion [71]. Thus, RAB7A could regulate MMP2 and cell migration on one hand by regulating AKT and NF-kB activity and on the other hand by regulating MT1-MMP recycling.

Importantly, we found that mutating the RAB7A Y183 amino acid changes the structure of the protein affecting the interaction with vimentin and, consequently, mutant proteins are not able to act as the wild-type. Indeed, for instance, RAB7A^Y183F^ is not able to rescue RAB7A activity on PAK1 and AKT after silencing and its expression does not cause nuclear translocation of NF-kB. The possible involvement of phosphorylation of this residue in these events needs further investigation.

Notably, we found that RAB7A depletion affects MMP2 activity but not abundance of pro and active MMP2 in the whole cell lysates. A possible explanation of these results could be that in Rab7-depleted cells there is a defective secretion of MMP2. This has been previously reported in cortactin silenced cells, where MMP2 abundance in cell lysates was not affected while abundance and activity in conditioned medium were decreased, suggesting that cortactin is important for MMPs secretion [72]. Importantly, it is known that RAB7A, cortactin and F-actin colocalize in circular dorsal ruffles and that RAB7A overexpression increases their formation [73]. Thus, these data suggest that RAB7A depletion could alter MMP2 secretion possibly through cortactin.

The link between RAB7A and MMPs could be relevant for potential clinical applications. The use of MMP inhibitors in clinical trials was unsuccessful possibly because of the lack of specificity of these drugs towards MMPs [74]. Failure of these inhibitors, which act by blocking the catalytic sites of the MMPs, was also related to the onset of musculoskeletal syndrome in patients [75]. MMPs not only have proteolytic functions degrading the extracellular matrix but they also associate to cell-surface receptors through their hemopexin-like C-terminal (PEX) domain. For example, pro-MMP2 interacts with integrin αvβ3, leading to increased VEGF (Vascular Endothelial Growth Factor) expression, stimulated by the activation of PI3K/AKT pathway, and to angiogenesis [76]. Thus, new therapeutic strategies are focusing on inhibitors that can prevent the binding of MMPs to cell surface receptors, such as, for instance, a synthetic peptide that blocks the MMP9 PEX domain leading to decreased cell migration of HT-1080 and MDA-MB-435 tumor cells [77]. Considering our data, another strategy could be represented by RAB7A inhibition that, reducing MMPs secretion and activity, could lead to a reduced activation of the PI3K/AKT pathway involved in tumorigenesis.

## 5. Conclusions

In conclusion, we proved that RAB7A influences AKT and PAK activity, regulating, also, several of their downstream effectors and impacting on the reorganization of vimentin filaments, which favor cell migration (Figure 8).

## Data Availability

The data presented in this study are available on request from the first author.

## Supplementary Materials

The following are available online at https://www.mdpi.com/article/10.3390/cancers13092220/s1, Table S1: Docking simulations (performed by Gramm-X followed by FireDock) between Vimentin model (Receptor) and RAB7A^WT^ whole model (ligand), Table S2: Docking simulations (performed by PatchDock followed by FireDock) between Vimentin model (Receptor) and fragments of RAB7A model (ligand), Table S3: Docking simulations (performed by Gramm-X followed by FireDock) between Vimentin model (Receptor) and mutant model (Y183H and Y183F) of RAB7A (ligand); Table S4: Results of Dynamut (folding stabilization of destabilization of 183 mutants); Table S5: Docking simulations (performed by PatchDock followed by FireDock) between Vimentin model (Receptor) and different models of RAB7A (ligand, downloaded by PDB-RCSB database), Table S6: Results of CABSflex, the correspondent fluctuation plot was shown in Figure 3 panel F. Original Western blots.
