# Supplementary material for: RAB7A Regulates Vimentin Phosphorylation through AKT and PAK
Source: Cancers (Basel). 2021 May 6;13(9):2220. doi: 10.3390/cancers13092220 (PMC8125308; doi:10.3390/cancers13092220)
Supplement: Supplementary file 1 [file cancers-13-02220-s001.zip › Original western blots.pdf]

Original gel/blots

Figure 1

b

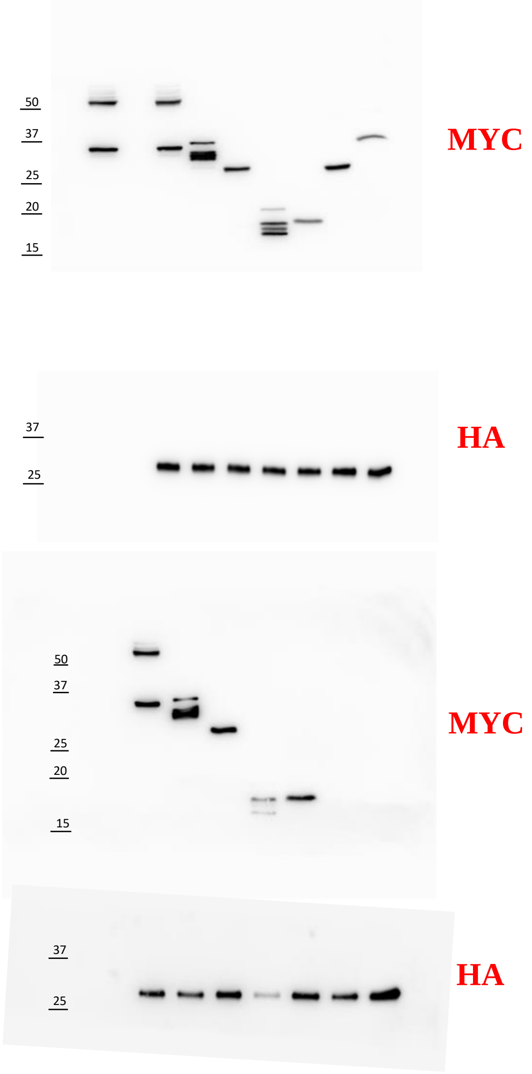

Figure 3

c

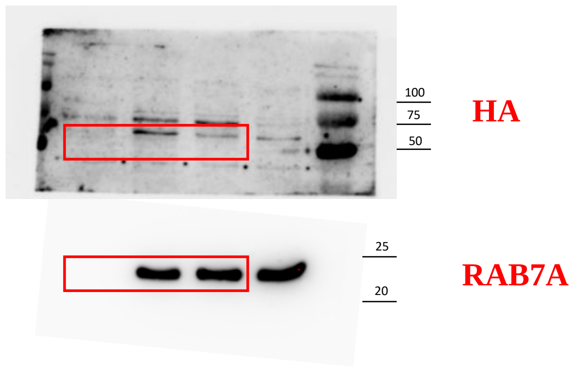

d

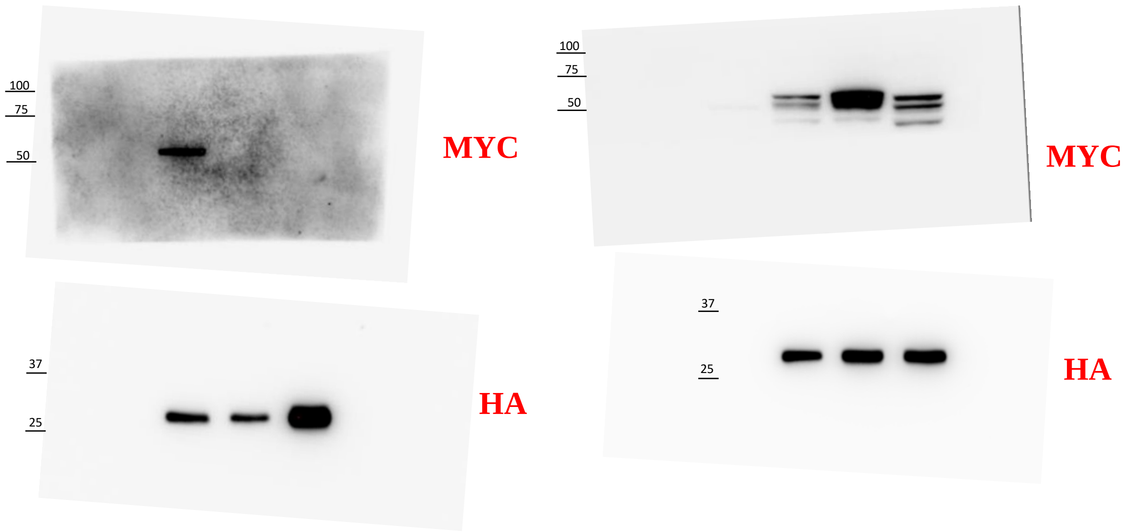

Figure 4

a

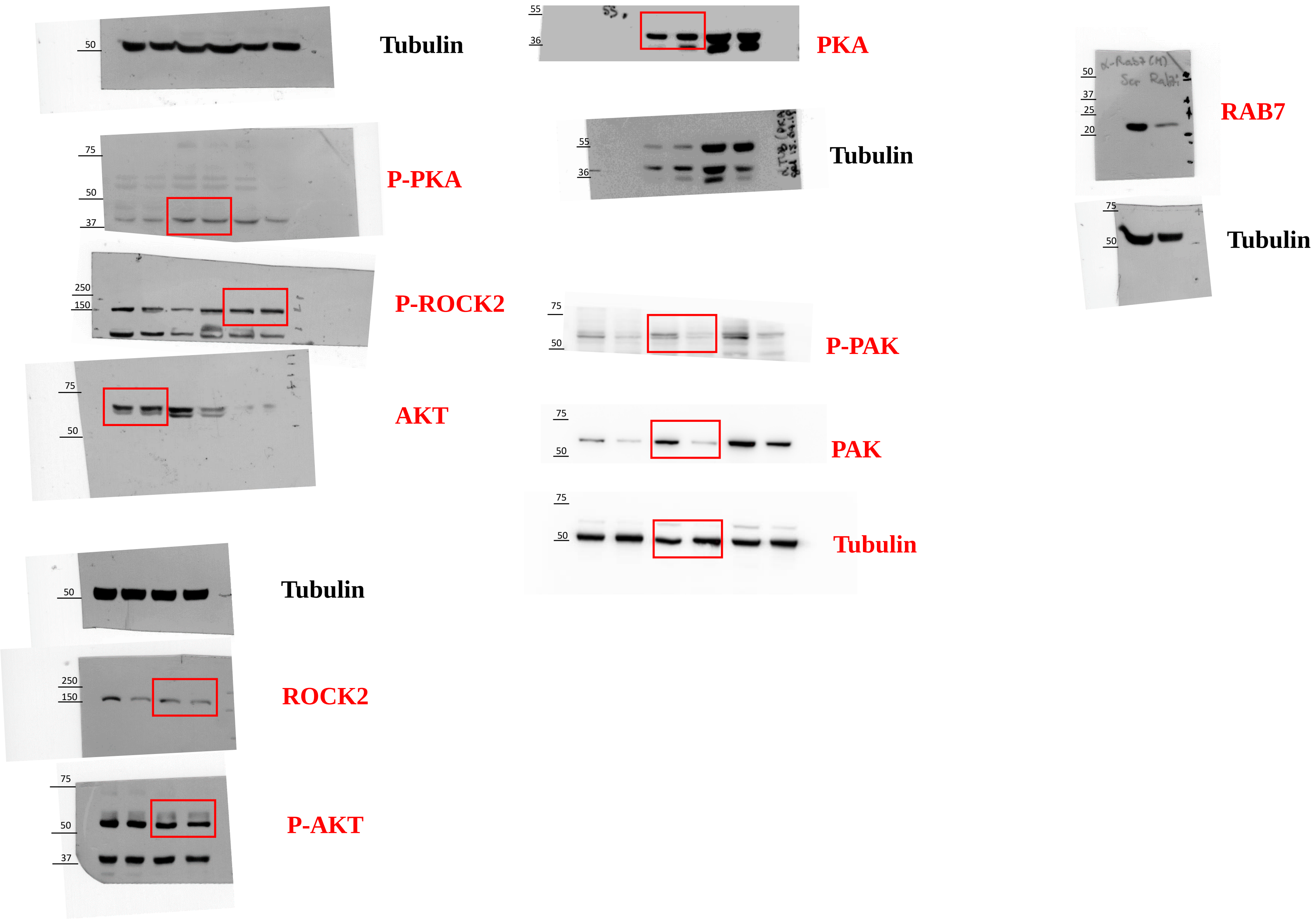

50
Tubulin
75
50
37
P-PKA
250
150
P-ROCK2
75
50
AKT
50
Tubulin
250
150
ROCK2
75
50
37
P-AKT
55
36
PKA
55
36
Tubulin
75
50
P-PAK
75
50
PAK
75
50
Tubulin
50
37
25
20
RAB7
75
50
Tubulin

Figure 4

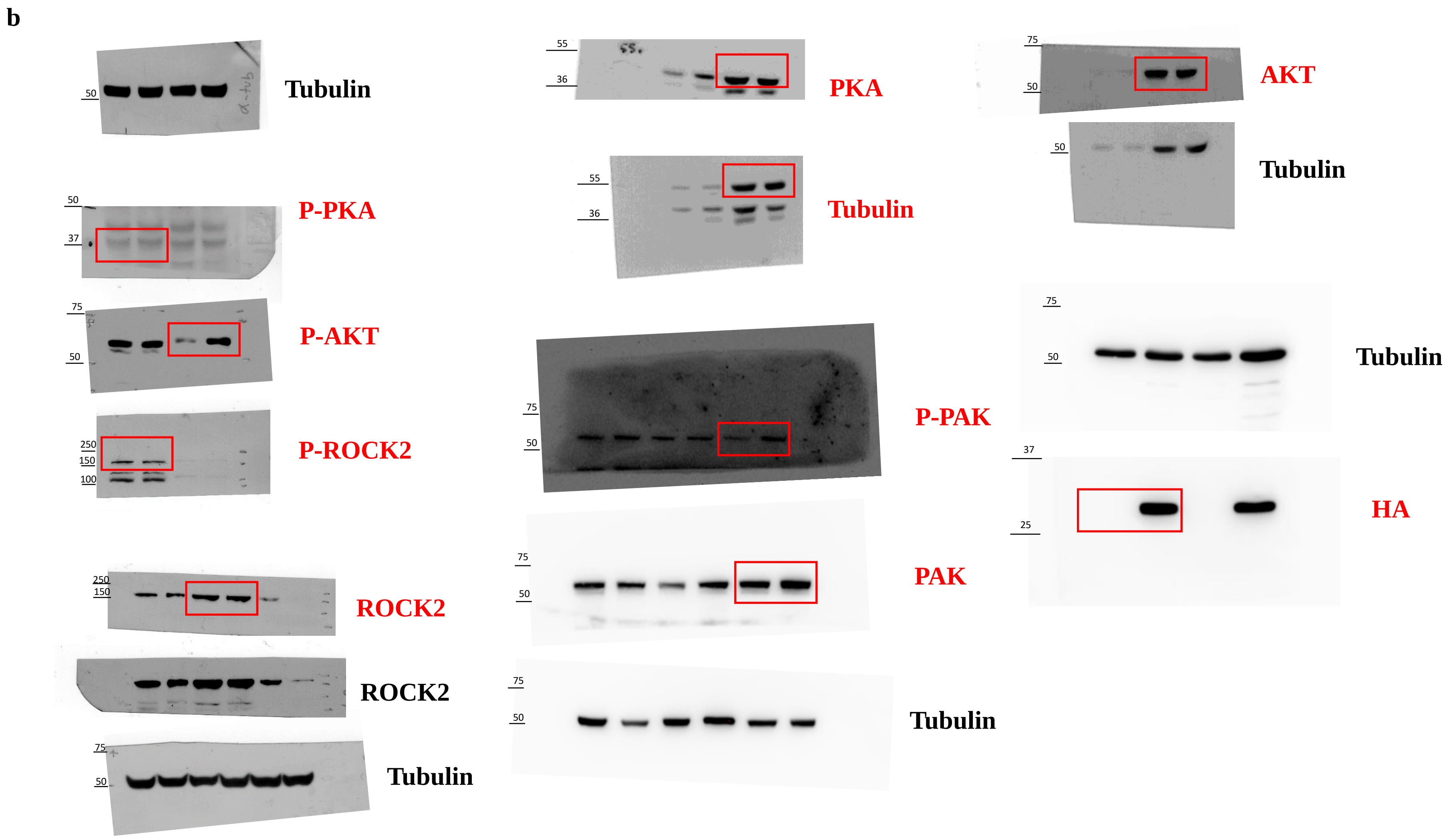
b
50
Tubulin
50
37
P-PKA
75
50
P-AKT
250
150
100
P-ROCK2
250
150
ROCK2
ROCK2
75
50
Tubulin
55
36
PKA
55
36
Tubulin
75
50
P-PAK
75
50
PAK
75
50
Tubulin
75
50
AKT
50
Tubulin
75
50
Tubulin
37
25
HA

Figure 4

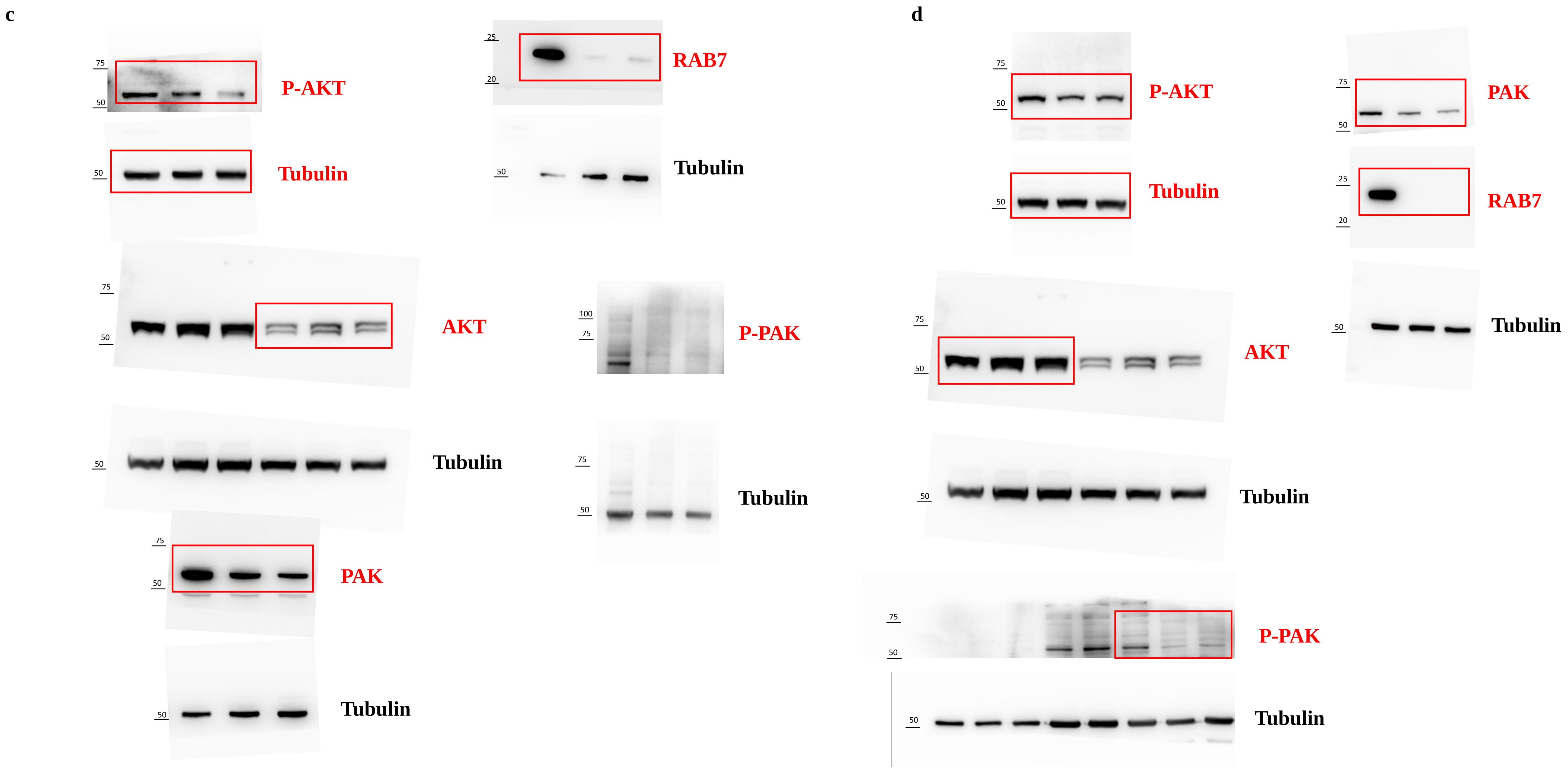
c
75
50
P-AKT
50
Tubulin
25
20
RAB7
50
Tubulin
75
50
AKT
100
75
P-PAK
50
Tubulin
75
50
Tubulin
75
50
PAK
50
Tubulin
d
75
50
P-AKT
50
Tubulin
75
50
PAK
25
20
RAB7
75
50
AKT
50
Tubulin
50
Tubulin
75
50
P-PAK
50
Tubulin

Figure 4

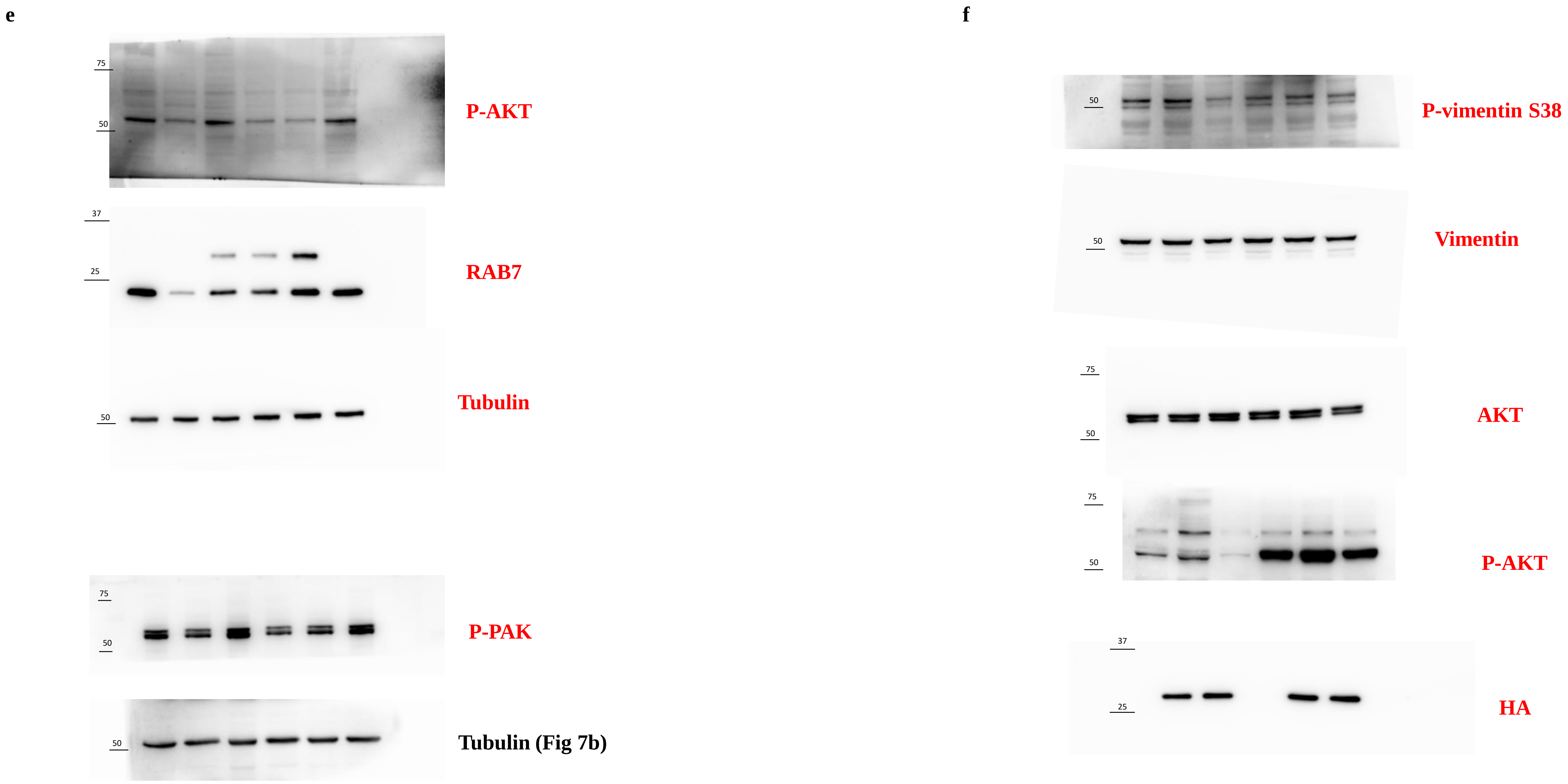
e
75
50
P-AKT
37
25
RAB7
50
Tubulin
75
50
P-PAK
50
Tubulin (Fig 7b)
f
50
P-vimentin S38
50
Vimentin
75
50
AKT
75
50
P-AKT
37
25
HA

Figure 5

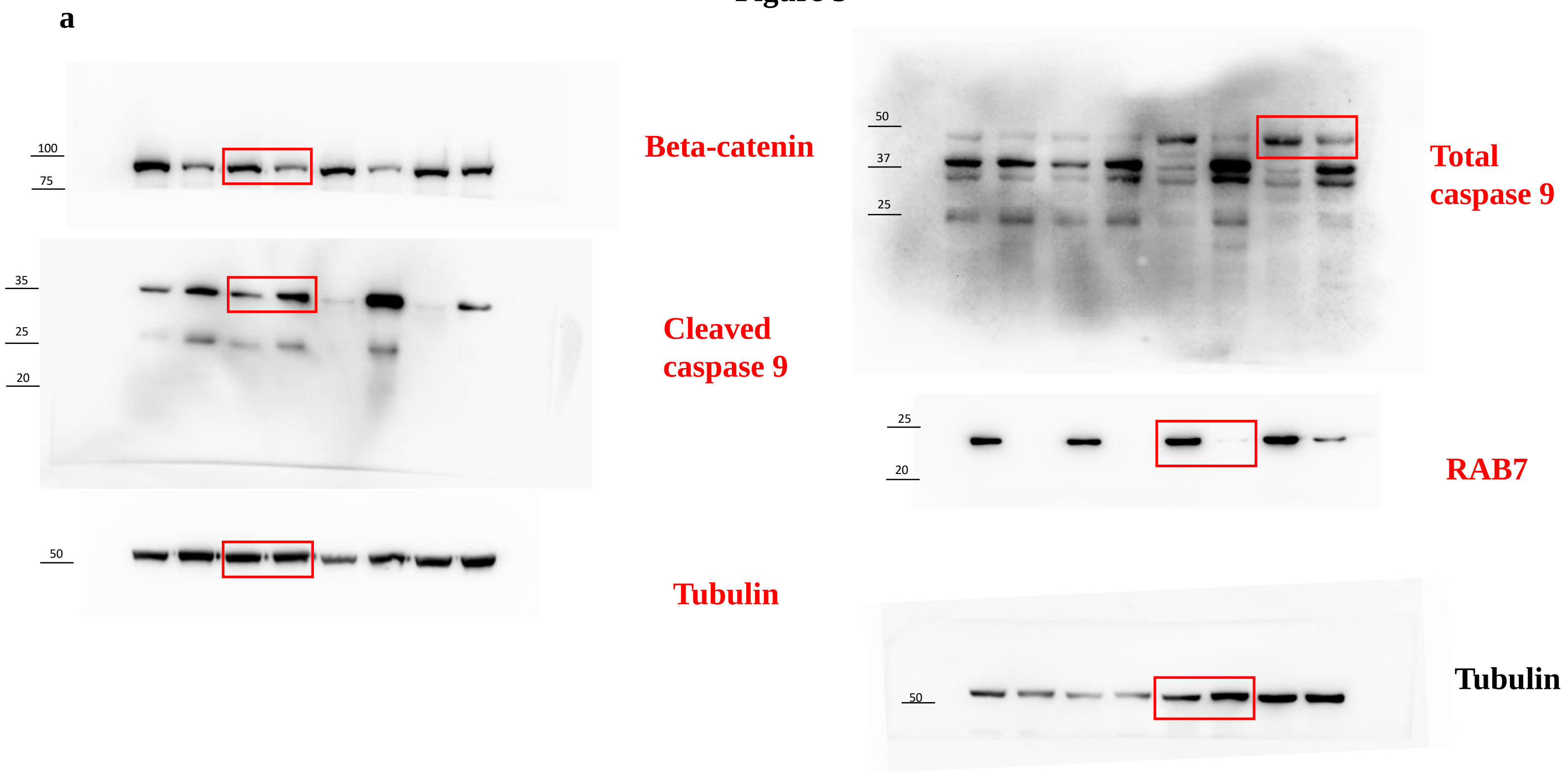
a
100
75
Beta-catenin
35
25
20
Cleaved caspase 9
50
Tubulin
50
37
25
Total caspase 9
25
20
RAB7
50
Tubulin

Figure 5

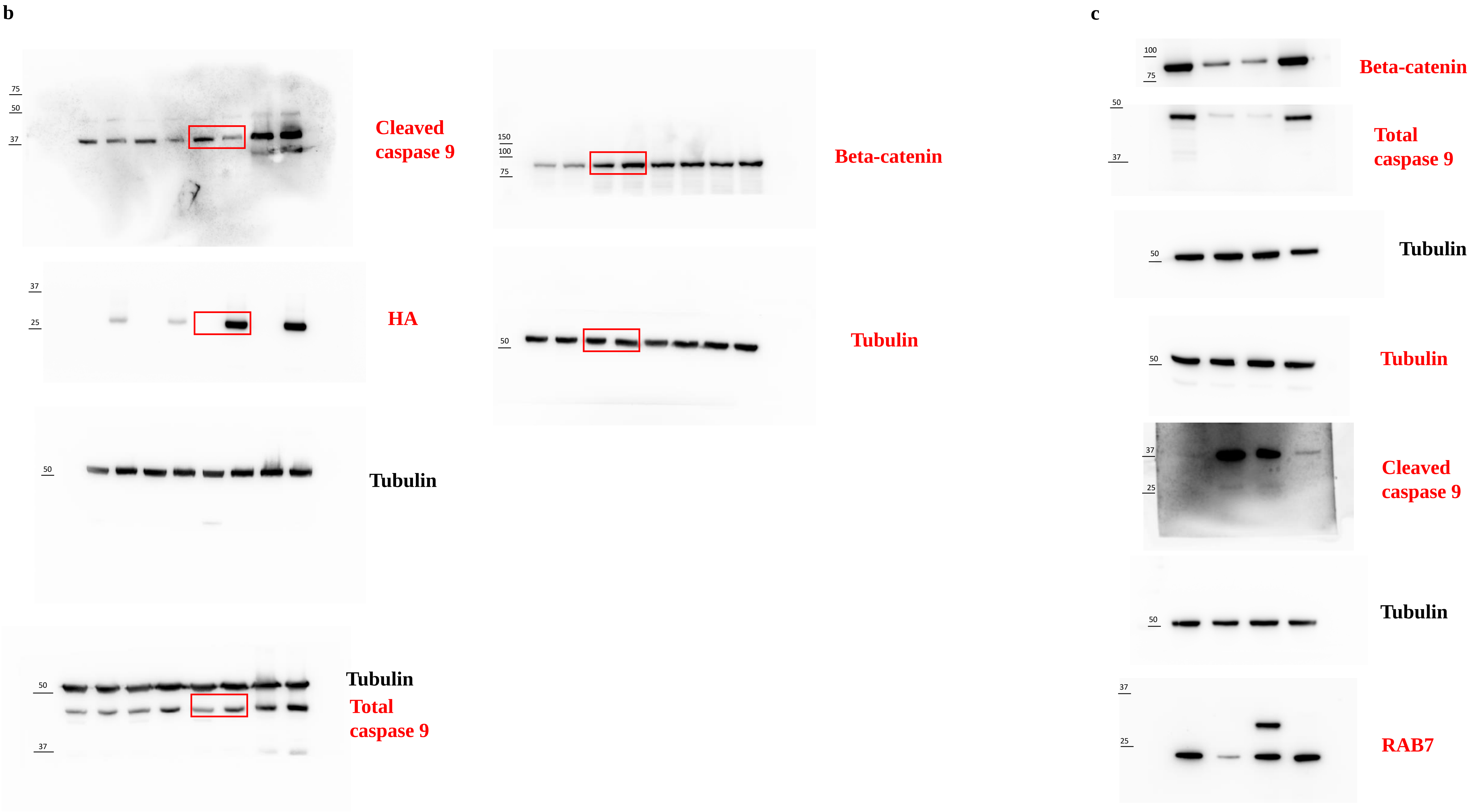
b
75
50
37
Cleaved caspase 9
37
25
HA
50
Tubulin
50
37
Tubulin
Total caspase 9
150
100
75
Beta-catenin
50
Tubulin
c
100
75
Beta-catenin
50
37
Total caspase 9
50
Tubulin
50
Tubulin
37
25
Cleaved caspase 9
50
Tubulin
37
25
RAB7

Figure 6

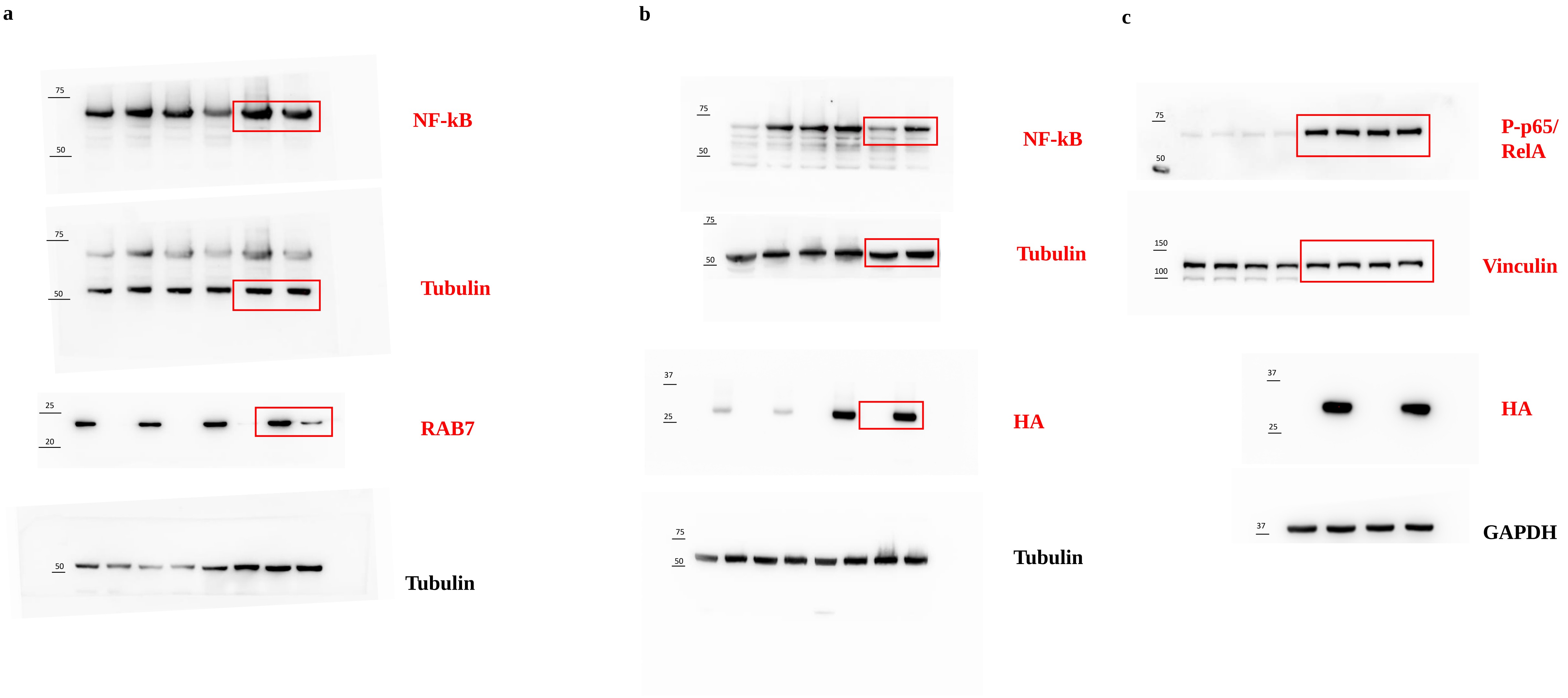

a
b
c
75
50
NF-kB
Tubulin
25
20
RAB7
Tubulin
37
HA
150
100
P-p65/
RelA
Vinculin
GAPDH

Figure 7

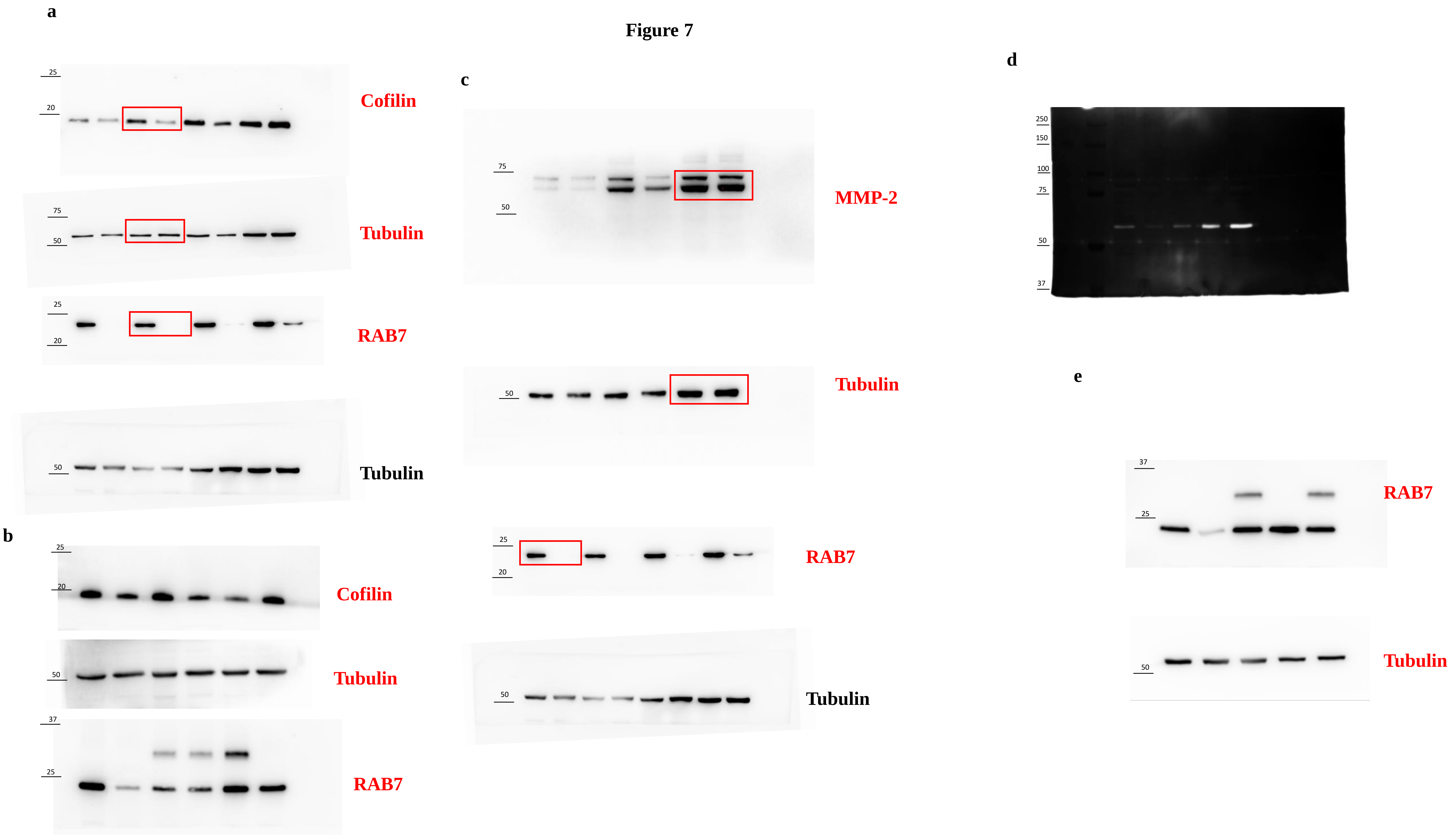
a
25
20
Cofilin
75
50
Tubulin
25
20
RAB7
50
Tubulin
b
25
20
Cofilin
50
Tubulin
37
25
RAB7
c
75
50
MMP-2
50
Tubulin
25
20
RAB7
50
Tubulin
d
250
150
100
75
50
37
e
37
25
RAB7
50
Tubulin
